# The potential contribution of aberrant cathepsin K expression to gastric cancer pathogenesis

**DOI:** 10.1007/s12672-023-00814-z

**Published:** 2024-06-10

**Authors:** Zhijun Feng, Lina Gao, Yapeng Lu, Xiaodong He, Jianqin Xie

**Affiliations:** 1 Jiangmen Central Hospital, No. 23, Haibang Street, Pengjiang District, Jiangmen, Guangdong China; 2 Laboratory Medicine Center, Lanzhou University Second Hospital, No. 82, Cuiyingmen, Chengguan District, Lanzhou, Gansu China; 3 Department of Anesthesiology, Lanzhou University Second Hospital, No. 82, Cuiyingmen, Chengguan District, Lanzhou, Gansu China; 4 The Second Clinical Medical College, Lanzhou University, No. 82, Cuiyingmen, Chengguan District, Lanzhou, Gansu China

**Keywords:** Cathepsin K, Gastric cancer, Cell invasion, Epithelial–mesenchymal transition, Immunosuppressive TME

## Abstract

The role of cathepsin K (CTSK) expression in the pathogenesis and progression of gastric cancer (GC) remains unclear. Hence, the primary objective of this study is to elucidate the precise expression and biological role of CTSK in GC by employing a combination of bioinformatics analysis and in vitro experiments. Our findings indicated a significant upregulation of CTSK in GC. The bioinformatics analysis revealed that GC patients with a high level of CTSK expression exhibited enrichment of hallmark gene sets associated with angiogenesis, epithelial–mesenchymal transition (EMT), inflammatory response, KRAS signaling up, TNFα signaling via KFκB, IL2-STAT5 signaling, and IL6-JAK-STAT3 signaling. Additionally, these patients demonstrated elevated levels of M2-macrophage infiltration, which was also correlated with a poorer prognosis. The results of in vitro experiments provided confirmation that the over-expression of CTSK leads to an increase in the proliferative and invasive abilities of GC cells. However, further evaluation was necessary to determine the impact of CTSK on the migration capability of these cells. Our findings suggested that CTSK has the potential to facilitate the initiation and progression of GC by augmenting the invasive capacity of GC cells, engaging in tumor-associated EMT, and fostering the establishment of an immunosuppressive tumor microenvironment (TME).

**Supplementary Information:**

The online version contains supplementary material available at 10.1007/s12672-023-00814-z.

## Introduction

Gastric cancer (GC) is a malignant neoplasm affecting the function of gastrointestinal tract, and it ranks among the most prevalent malignancies globally. The growing body of research on cancer-related genomics has led to an increased focus on investigating the genetic attributes underlying the development and occurrence of GC. Although molecular subtypes, pioneered by The Cancer Genome Atlas (TCGA) [1], provide more guidance for the clinical diagnosis and treatment of patients with GC, there is still a long way to go. In gastritis patient with or without *Helicobacter pylori* (*H. pylori*) infection that has evolved to GC, changes in genomics and the microenvironment play vital roles [2]. Abnormal gene expression not only facilitates the acquisition of carcinogenic abilities by healthy normal cells directly, but it also plays a role in the development of the tumor microenvironment (TME), thereby indirectly conferring tumorigenic potential to gastric mucosal cells.

CTSK, a cysteine protease found in lysosomes, is expressed in Osteoclasts, which play a crucial role in bone remodeling [3]. CTSK is associated with tumor invasion, progression, lymph node metastasis, and bone metastasis in the context of malignancy [4–9]. Additionally, CTSK serves as a potential therapeutic target for patients with breast or prostate cancer who are at a heightened risk of bone metastasis [10, 11]. In our previous study, we presented findings from a bioinformatics analysis indicating the potential significance of the CTSK gene in the prognostic evaluation of GC patients [12]. However, we did not investigate the impact of CTSK on GC cell proliferation and differentiation. To date, there is a dearth of literature on the potential roles and underlying mechanisms of CTSK in the pathogenesis and progression of GC, necessitating further investigation.

In this study, we conducted an evaluation of the disparities in CTSK expression between cancerous and healthy normal tissues in patients with GC. This evaluation was based on gene expression datasets obtained from reputable public databases such as Oncomine [13], Tumor Immune Estimation Resource (TIMER) [14], and Gene Expression Omnibus (GEO) [15]. Additionally, we performed immunohistochemical (IHC) analyses to assess CTSK expression in various types of GC and their corresponding adjacent noncancerous tissues. Furthermore, we stratified the candidate samples from GEO database into lower- and higher-CTSK groups based on a threshold determined through survival analyses, in order to gain further insights into the potential mechanism of CTSK involvement in GC progression. Additionally, we conducted gene set enrichment analysis (GESA) to compare the two groups exhibiting varying levels of CTSK expression. Furthermore, we employed the CIBERSORT method to evaluate the degree of immune cell infiltration in the TME. Finally, through in vitro experiments, we assessed the effects of CTSK knockdown and over-expression on GC cell proliferation, migration, and invasion. These findings not only shed light on the potential significance and molecular mechanisms of CTSK in the progression of GC, but also elucidate the interplay between CTSK and the tumor-immune response at the gene-cell level.

## Materials and methods

### Gene expression analyses

Using the TIMER and Oncomine databases, we conducted a comprehensive analysis of the expression levels of CTSK in multiple cancer types. The findings of this investigation were visually represented using the visualization tools provided by the respective databases. Additionally, we obtained three gene expression profiles (GSE2669, GSE54129, and GSE65801 [16]) from the GEO database to compare the expression levels of CTSK between tumor tissues and healthy normal tissues in GC. The visualization of the results was performed using the ggplot2 package [17] in the R software (Version 4.1.0, https://www.r-project.org/). The GSE2669 dataset included samples with six distinct pathological types: normal gastric tissue, chronic gastritis (CG), intestinal metaplasia (IM), and the intestinal, diffuse, and mixed types of GC. It is noteworthy that, to mitigate the influence of ethnic variables on the analysis outcomes, we exclusively opted for research cohorts from the Asian population affected by gastric cancer when scrutinizing gastric cancer-related data in the GEO database. A comprehensive account of the data screening procedure can be found in Supplementary Material 1.

### IHC staining and clinical characteristics analysis

A total of 90 GC patients who underwent radical resection, along with their corresponding adjacent tissues, were gathered for analysis. These tissues were then consolidated into a tissue chip. Immunohistochemical (IHC) staining was employed to examine the variations in CTSK expression between tumor and normal tissues. The CTSK antibody (EPR19992) utilized in this study was procured from Abcam Company and obtained by Lanzhou KEBAO Biotechnology Co., LTD. The study was carried out strictly following the Declaration of Helsinki and was approved by the Medical Ethics Committee of Lanzhou University Second Hospital (2021A-048). Informed consent was taken from all patients. The IHC staining procedure was conducted using a concentration ratio of 1:300, following the SP method as described in “Experimental Techniques in Molecular Biology”. The experimental details, including the equipment, reagents, and operational steps, can be found in Supplementary Material 1. The assessment of the ultimate IHC staining score was delineated in the subsequent manner: the staining intensity score was categorized as follows: 0, denoting negativity; 1, indicating weak staining; 2, signifying moderate staining; 3, representing strong staining. The score corresponding to the percentage of positive cells was defined as follows: 0, less than 5%; 1, ranging from 5 to 25%; 2, spanning from 26 to 50%; 3, encompassing 51% to 75%; 4, exceeding 75%. The staining index, ranging from 0 to 12, was determined by multiplying the staining intensity score with the positive area score, where a score of 0–6 indicated low expression and a score of 7–12 indicated high expression. Pathological diagnoses were conducted by a minimum of two pathologists.

To further elucidate the association between CTSK and the clinical pathological characteristics of GC patients, this study employed two approaches. Firstly, the clinical data of 90 GC patients were utilized to assess the correlation between CTSK and various clinical parameters including GC TNM stage, depth of invasion (T-staging), lymph node metastasis (N-staging), distant metastasis (M-staging), differentiation degree, and age (comparing the > 60-year-old group with the ≤ 60-year-old group). Secondly, the clinico-pathological data of 1215 GC patients from the GEO database were analyzed, specifically the GSE62254 dataset [18, 19] comprising 300 GC cases, and the GSE26253 dataset [19, 20] comprising 432 GC cases, and and the GSE84437 [19] dataset comprising 483 GC cases. Furthermore, utilizing the STAD data available in the TCGA database, an investigation was conducted to examine the variation in CTSK expression across different stages of GC as defined by the TNM classification system. The Supplementary Material 1 provides detailed explanations of the procedures involved in data retrieval, standardization, extraction, and analysis. The outcomes were visually represented through a combination of box and dot plots, with statistical significance determined at a threshold of *p* < 0.05.

### GSEA

GSEA was conducted on the two cohorts (GSE26253, GSE62254) utilizing the clusterProfiler package [21]. Initially, the candidate samples were categorized into low- and high-CTSK expression groups based on the survival analysis threshold in our previously published data [12]. Subsequently, the fold change (FC) in gene expression between the two groups was computed using the limma package [22]. Following these procedures, the gene symbols were converted to Entrez Identifications (EntrezID), and an integrated analysis was performed with the hallmark gene sets (Version 7.1) obtained from the Molecular Signatures Database [23]. Subsequently, the outcomes were depicted in the form of a bubble chart, wherein a adjusted *p* value < 0.05 was observed.

### Prognositic evaluation and estimated immune cell types in TME with CIBERSORT

The evaluation of the proportions of diverse immune cells within the TME was conducted using CIBERSORT [24]. The leukocyte signature matrix (LM22), comprising 547 genes that differentiate 22 distinct phenotypes of human hematopoietic cells, including 7 T-cell types, naive and memory B cells, plasma cells, natural killer (NK) cells, and myeloid cell subsets, is widely employed for estimating the fractions of immune cell types [25]. We employed the CIBERSORT algorithm to integrate LM22 with the normalized gene expressions of the GSE26253 and GSE62254 datasets, resulting in the determination of immune cell infiltration levels for each dataset. Subsequently, we conducted an assessment of the effects of LM22-labeled immune cells on the overall survival (OS) and disease-free survival (DFS) rates in patients diagnosed with GC, based on the key findings from the survival analysis as previously described. Additionally, we examined the disparities in immune cell infiltration levels between the cohorts exhibiting lower and higher expression of CTSK. Subsequently, we assessed the impact of infiltrated immune cells on the survival outcomes of GC patients.

### GC cell line culture

The human GC cell lines AGS, HGC27, MKN45, NCI-87, and the human gastric mucosal normal cell line GES1 were preserved by the Key Laboratory of Digestive System Tumors, Lanzhou University Second Hospital (Lanzhou, China). Initially, the basal expression of CTSK was calculated in the above-mentioned cells, using the GES1 cell line as the reference group. Two cell lines, AGS cells with higher CTSK expression and MKN45 cells with lower CTSK expression, were selected for further experiments. Subsequently, lentivirus obtained from Shanghai Jikai Company was employed to construct GC cells with CTSK knockdown/over-expression. We chosen an multiplicity of infection (MOI) of 15 for AGS cells and 30 for MKN45 cells. The cell concentration of all samples was approximately 2 × 10^4^ during transfection. After 24 h, the culture medium containing the virus was removed and replaced with fresh culture medium, and the culture was subsequently maintained. Fluorescence was observed under a microscope 48 h after infection (Figures S2 and S3 in Supplementary Material 1). Following these procedures, we successfully acquired four distinct cell lines essential for our research: AGS cell (exhibiting higher basal CTSK expression), sh-CTSK AGS cell (AGS cell line with CTSK knockdown), MKN45 cell (displaying lower basal CTSK expression), and EO-CTSK MKN45 cell (MKN45 cell line with CTSK over-expression) (Figures S4 and S5 in Supplementary Material 1). Henceforth, we shall collectively refer to these cell lines as “candidate cells”. Real-time quantitative PCR (RT-qPCR) was employed during this phase, and the methodologies for cell line cultivation, mRNA extraction, PCR array analysis, viral transfection, and assessment of transfection efficiency were elucidated in Supplementary Material 1. For all subsequent experiments, a minimum of three independent replicates was done.

### CCK-8 assay

The Cell Counting Kit-8 (CCK-8) test was conducted to evaluate cell viability using a CCK-8 kit (Abcam, USA). The candidate cells were cultured in a growth medium in triplicate wells (2 × 10^3^ cells/well) of a 96-well plate for 48 h. Subsequently, the medium was substituted with DMEM/10% FBS containing 10% CCK-8 solution and incubated at 37 °C for 1 h in the absence of light. The plate was subsequently subjected to analysis using a microplate reader manufactured by TECAN in Switzerland, with the purpose of quantifying the optical density at a wavelength of 450 nm (OD450) over a period of 24 h, 48 h, and 72 h.

### Colony formation assays

Candidate cells were seeded at a density of 800 cells per well in six-well plates. Subsequently, the cells were cultured in RPMI-1640 media supplemented with 10% fetal bovine serum (FBS) for approximately one weeks under incubation conditions of 37 °C and 5% CO_2_. Following this incubation period, the colonies were rinsed and subjected to treatment with 100% methanol and 0.2% crystal violet for a quarter of the total time. After two subsequent washes with phosphate-buffered saline (PBS), the colonies were visualized and captured using a microscope.

### Apoptosis detection

The Annexin V-FITC apoptosis detection kits were procured from Yeasen (Shanghai, China) and utilized for the assessment of cellular apoptosis. Briefly, the candidate cells were harvested using 0.25% trypsin and subsequently collected in a centrifuge tube. Following centrifugation (500×*g*/min, 5 min, 4 °C), the supernatants were discarded, and the plates were resuspended in 500 μL of binding buffer. Subsequently, Annexin V-FITC reagents (5 μL) and PI solution (10 μL) were introduced into the cells. After incubation in darkness for a quarter, the cells were subjected to flow cytometry assays.

### Wound healing assay

The wound healing test was conducted to demonstrate the impact on cell migration. Candidate cells were cultured in a 6-well plate and allowed to achieve a confluence of 90–100% by the second day. A 1.0 mL pipette tip was utilized to create a straight line on the cell surface, thereby generating the wound area. The detached cells were eliminated, and viable cells were maintained in FBS-free DMEM. The initial point was marked on the plate's bottom using a marking pen. Wound images were captured using a microscope at 0 h and 24 h, and the wound gap area was quantified using ImageJ software.

### Transwell assay

In this study, the Matrigel (DMEM 1:8 dilution, Corning, USA) was applied as a precoating in a transwell chamber (Corning, USA) and incubated at 37 °C for a duration of 2 h prior to the commencement of the experiment. A total of 5 × 104 cells in serum-free DMEM were introduced into the upper wells of the chamber, while DMEM containing 20% FBS was added to the lower chamber. The chambers were then incubated for a period of 24 h within an incubator. Subsequently, the chambers were fixed using 4% PFA and stained with 0.5% crystal violet. The Matrigel and cells present in the upper chamber were eliminated using a cotton swab. The optical microscope was employed to capture images of the invading cells, which were subsequently analyzed using Image J software.

### Statistical analysis

The distribution of gene expression was determined using the D'Agostino-Pearson normality test, while variance homogeneity was assessed using the F-test. If the gene expression data exhibited normal distribution and uniform variances, the statistical significance between groups was determined using the Student’s t-test. Alternatively, the Mann Whitney-Wilcoxon test was employed if these assumptions were not met. Survival curves were determined through Kaplan–Meier analysis and log-rank tests. Statistical significance was considered present when *p* < 0.05.

## Results

### Gene expression analysis

**Fig. 1:**
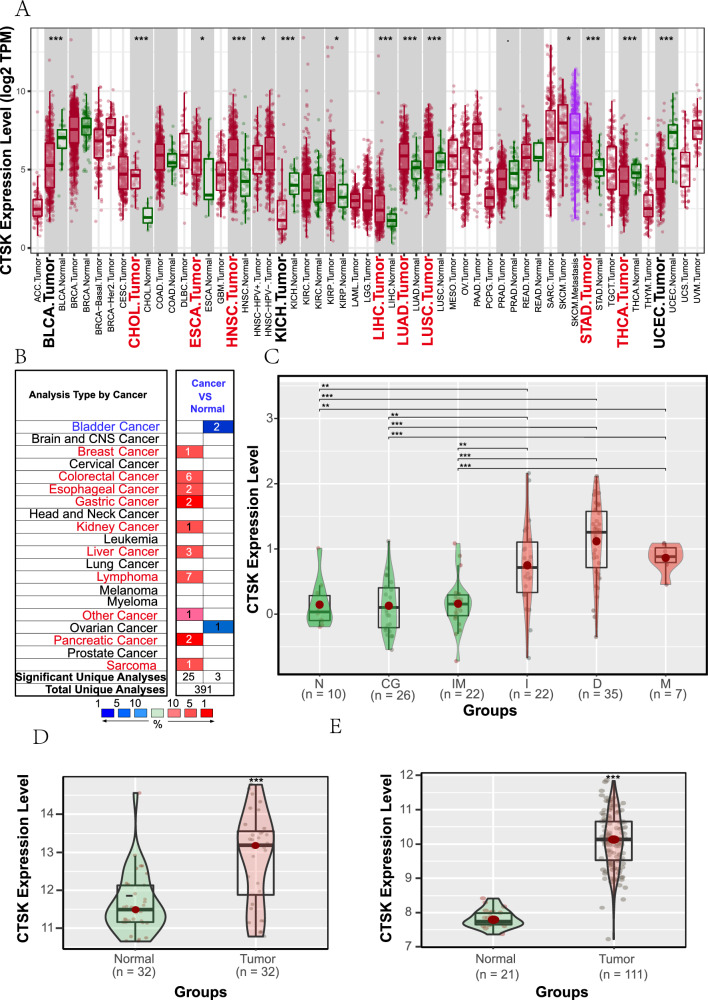
Expression levels of CTSK in various human cancers. **A** The pan-cancer expression levels of CTSK gene from the TIMER database, and tumor types exhibiting higher CTSK expression levels in tumor tissues compared to normal tissues are highlighted in bold red font. **B** The results of comprehensive analysis of pan-cancer expression of CTSK gene from Oncomine database. There should be small squares behind each cancer type, where red represents high CTSK expression in the current cancer type compared to normal tissue and blue represents low expression. The numbers in the squares represent the number of datasets with high and/or low CTSK expression in the current cancer type. **C** Expression levels of CTSK in healthy normal samples (N), samples with chronic gastritis (CG), samples with intestinal metaplasia (IM), samples with intestinal (I), diffuse (D), and mixed (M) type of gastric cancer from GSE2669 dataset (light green represents non-tumor tissues, and light red represents tumor tissues). **D**, **E** Expression levels of CTSK in normal tissues from healthy people and tumor tissues from patients with gastric cancer in GSE65801 and GSE54129 dataset, respectively (light green represents non-tumor tissues, and light red represents tumor tissues). **F** The examination of genotypes revealed that the wild-type CTSK remained prevalent among the majority of gastric cancer patients from the TIMER database. *p < 0.05, **p < 0.01, ***p < 0.001, ****p < 0.0001

In comparison to healthy normal tissues, an elevated expression of CTSK was observed in GC tissues within the TIMER and Oncomine databases. Additionally, a heightened level of CTSK was detected in esophageal, kidney, and liver carcinoma tissues (Fig. 1A, B). By utilizing the GSE2669 dataset, our analysis revealed a significant up-regulation of CTSK in all three distinct subtypes of GC. However, in comparison to normal tissues, its expression level remained relatively stable in non-cancerous gastric lesions. This finding further emphasizes the oncogenic characteristics associated with CTSK (*p* < 0.05, Fig. 1C). The analysis findings of the GSE65801 and GSE54129 datasets revealed a significant up-regulation of CTSK in GC tissues when compared to normal tissues, with a statistically significant difference (Fig. 1D for GSE65801, Fig. 1E for GSE54129).

### IHC staining and clinical characteristics analysis

**Fig. 2:**
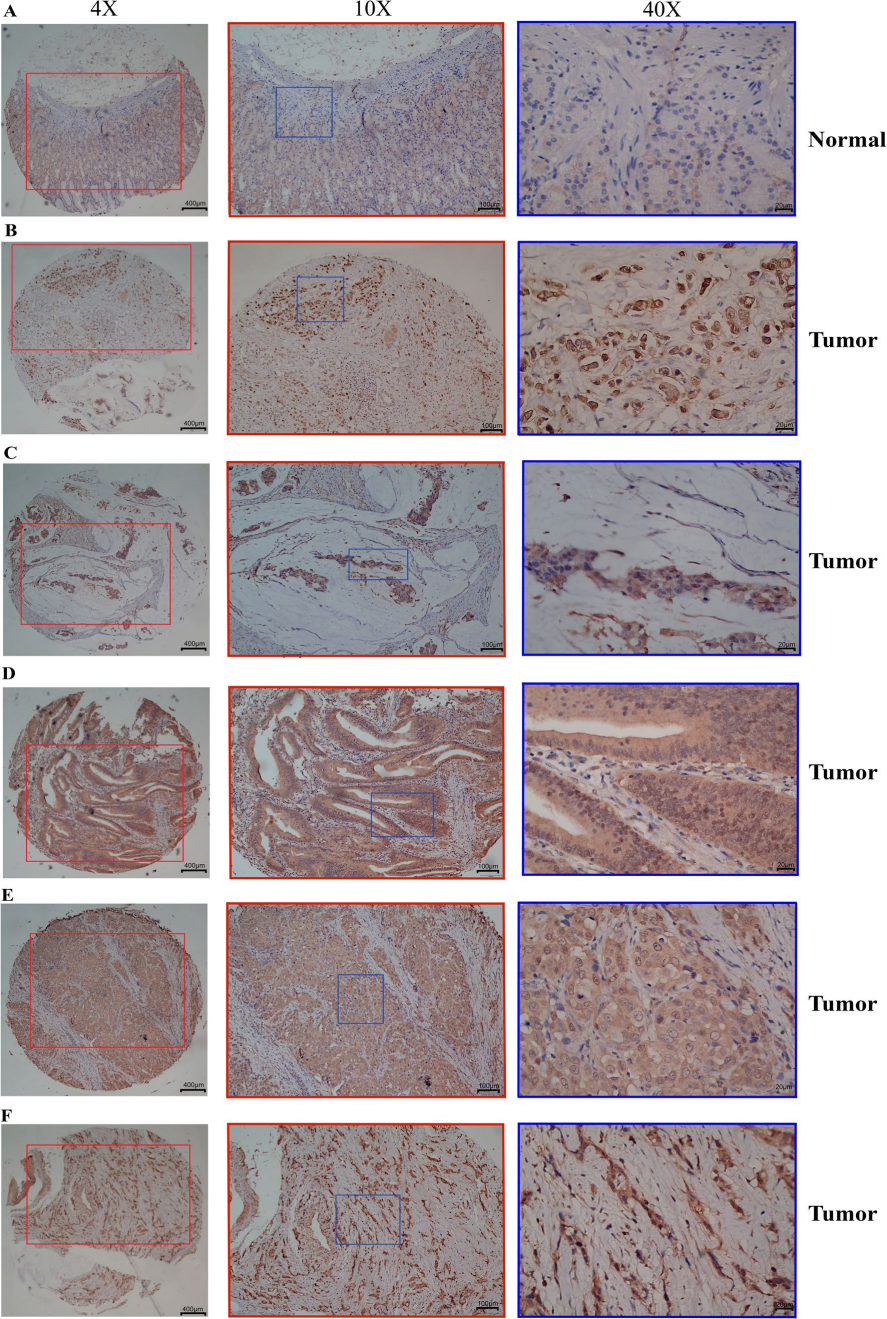
Representative images of immunohistochemical staining for CTSK in GC tissues and para-carcinoma tissues. The first column corresponds to a magnification of ×4, the middle column represents a magnification of ×10 (indicated by the red box, which aligns with the red box line in the first column), and the third column depicts a magnification of ×40 for the IHC image (the blue box denotes the ×40 magnification region, which coincides with the marked position in the middle column). **A** Shows the IHC results from a para-carcinoma tissues, and **B** to **F** for GC tissues. Nuclei were stained in blue, and positively expressed CTSK located in the cells was indicated with a pale yellow–brown or brown color

Based on the IHC findings, it was observed that CTSK was localized within the cytoplasm of both normal cells and GC cells, with a notable up-regulation observed in tumor tissues (*p* < 0.05, Table 1). Figure 2 displays the representative IHC staining outcomes, whereas Figure S1 in Supplementary Material 2 illustrates the comprehensive staining of the tissue microarray. Out of the 90 GC patients examined, it was observed that accurate scoring could not be obtained for 16 cases of normal tissues and 5 cases of cancer tissues due to issues such as poor staining, fixation, or degradation of the stain during the interval period. Among the 85 GC samples that were successfully scored, 5 cases were diagnosed as “mucinous adenocarcinoma”. In this study, we conducted a comprehensive analysis of the clinicopathological characteristics of patients with GC and examined the relationship between the efficacy of CTSK expression and these characteristics (Table 1). The findings indicated that there was a significant increase in the expression of CTSK in GC patients with lymph node metastasis compared to those without lymph node metastasis (p < 0.05, Table 1). Furthermore, an analysis of CTSK expression in GC patients with varying degrees of differentiation revealed a significant up-regulation in poorly differentiated GC tissues (p < 0.05, Table 1). These results provide additional evidence supporting the potential role of CTSK in promoting unfavorable prognostic characteristics in patients with GC.

**Table 1:** IHC staining and clinical characteristics analysis for gastric cancer patients

	Num	CTSK		X^2^	*p* value
		High	Low		
Tissues type					
Normal	74	20 (27.0%)	54 (73.0%)	9.186	**
Tumor	74	38 (51.4%)	36 (48.6%)		
TNM staging					
I	13	5 (38.5%)	8 (61.5%)		
II	29	16 (55.2%)	13 (44.8%)	1.003	*ns*
III	31	17 (54.8%)	14 (45.2%)	0.983	*ns*
IV	12	6 (50.0%)	6 (50.0%)	0.337	*ns*
T-staging					
T1	8	5 (62.5%)	3 (37.5%)		
T2	16	8 (50.0%)	8 (50.0%)	0.336	*ns*
T3	41	18 (43.9%)	23 (56.1%)	0.93	*ns*
T4	20	13 (65.0%)	7 (35.0%)	0.016	*ns*
N-staging					
N0	22	7 (31.8%)	15 (68.2%)	4.73	*
N1–3	63	37 (58.7%)	26 (41.3%)		
M-staging					
M0	73	38 (52.1%)	35 (47.9%)	0.017	*ns*
M1	12	6 (50.0%)	6 (50.0%)		
Age					
> 60 years	36	18 (50.0%)	18 (50.0%)	0.078	*ns*
≤ 60 years	49	26 (53.1%)	23 (46.9%)		
Grade of differentiation					
Low-	41	24 (58.5%)	17 (41.5%)	4.023	*
Moderate-	34	12 (35.3%)	22 (65.7%)		
High-	5	4 (80.0%)	1 (20.0%)	3.601	*ns*

*Num.* number of gastric cancer patients, *TNM staging* tumor node metastasis staging, *T-staging* primary tumor staging, *N-staging* lymph node metastasis staging, *M-staging* distant metastasis staging, *ns* no significance

*p < 0.05, **p < 0.01, ***p < 0.001, ****p < 0.0001

**Fig. 3:**
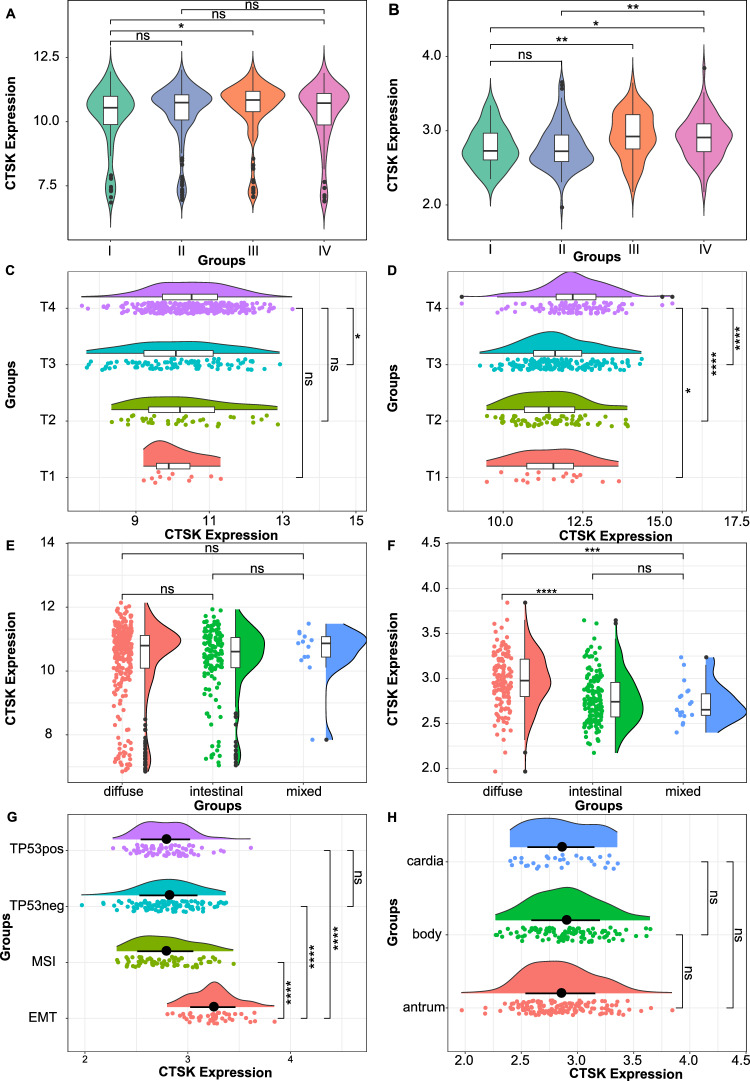
Analysis of the correlation between CTSK and clinical characteristics of patients with GC patients based on public gene expression databases. **A**, **B** Are the differences in CTSK expression levels between different clinical TNM stages (TNM stage: I, II, III, IV) in the data sets GSE26253 (**A**) and GSE62254 (**B**), **C**, **D** are the difference in CTSK expression level between different degrees of tumor invasion depth (T) stages (T-staging: T1, T2, T3, T4) in the data sets GSE84437 (**C**) and TCGA (**D**). **E**, **F** Are the difference in CTSK expression level between different Lauren types in the data sets GSE26253 (**E**) and GSE62254 (**F**). **G** Is the difference in CTSK expression level between different molecular subtypes (EMT, known as MSS: microsatellite stability, MSI: microsatellite instability, TP53pos: the pathway of tumor protein 53 activated, and TP53neg: loss of the function of tumor protein 53) in the data set GSE62254 (**G**). **H** Is the difference in CTSK expression levels in different tumor sites in the data set GSE62254 (**H**). *p < 0.05, **p < 0.01, ***p < 0.001, ****p < 0.0001

To better elucidate the correlation between the CTSK gene and clinicopathological characteristics in patients with GC, we expanded the sample size by utilizing publicly available databases. Figure 3A, B depict the disparities in CTSK expression levels across various clinical TNM stages in datasets GSE26253 (Fig. 3A) and GSE62254 (Fig. 3B). The findings indicate a significant elevation in CTSK expression among GC patients with advanced TNM stages compared to those with earlier TNM stages. Similarly, Fig. 3C, D represents the discrepancies in CTSK expression levels across different tumor invasion depth (T) stages (T-staging: T1–4) in GSE84437 datasets (Fig. 3C) and STAD data from TCGA (Fig. 3D). The results demonstrate a noteworthy increase in CTSK expression levels among GC patients with higher T-staging. The expression level of CTSK was assessed in various Lauren subtypes of GC using two cohorts obtained from the GEO database (GSE26253 and GSE62254). The findings from GSE26253 dataset indicated no significant disparity in CTSK expression among patients with diffuse, intestinal, and mixed GC (Fig. 3E). Conversely, the results from GSE62254 dataset demonstrated a significantly elevated expression of CTSK in patients with diffuse GC patients compared to intestinal or mixed GC (Fig. 3F). Molecular typing analysis revealed a high expression of CTSK in GC patients exhibiting the epithelial–mesenchymal transition (EMT) phenotype (Fig. 3G), while no significant variation in CTSK expression was observed among GC tissues from different anatomical sites (Fig. 3H). These pieces of evidence strongly suggest a close association between CTSK and unfavorable prognostic factors in GC, including TNM stage, t stage, diffuse type, and EMT molecular typing.

### GSEA

**Fig. 4:**
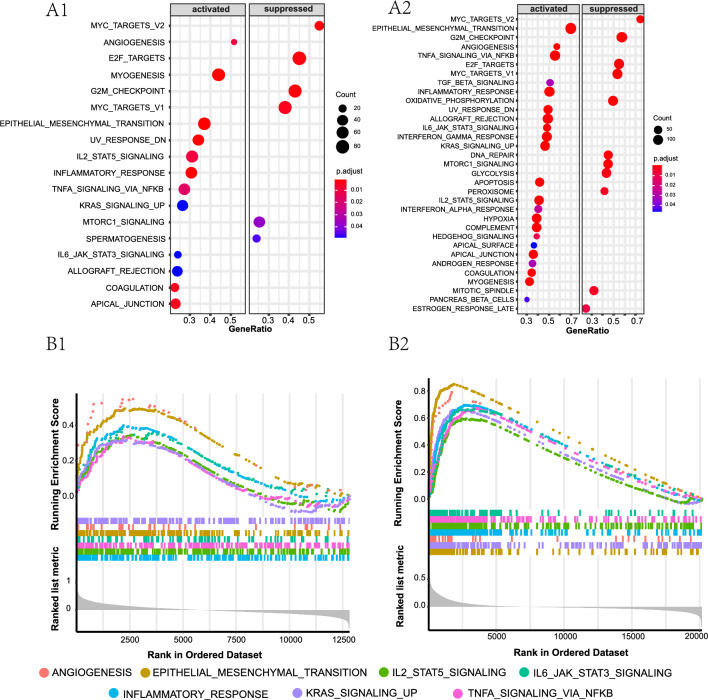
The results of gene-set enrichment analysis (GSEA) between the low and high-CTSK expression groups. **A1**, **A2** Total results of hallmark gene sets from GSE26253 (**A1**) and GSE62254 (**A2**) datasets. **B1**, **B2** Six of the co-enriching hallmark gene sets in GSE26253 (**B1**) and GSE62254 (**B2**) datasets. ANGIOGENESIS (genes up-regulated during the formation of blood vessels), EPITHELIAL–MESENCHYMAL TRANSITION (genes defining epithelial–mesenchymal transition), IL2-STAT5 SIGNALING (genes up-regulated by STAT5 in response to IL2 stimulation), IL6-JAK-STAT3 SIGNALING (genes up-regulated by IL6 via STAT3), INFLAMMATORY RESPONSE (genes defining inflammatory response), KRAS SIGNALING UP (genes up-regulated by KRAS activation), TNFA SIGNALING VIA NFKB (genes regulated by NF-κB in response to TNFα). *IL2* interleukin 2, *STAT* signal transducer and activator of transcription, *IL6* interleukin 2, *JAK* Janus Kinase

The GSEA results comparing the low and high-CTSK expression groups in the two GEO cohorts (GSE26253 and GSE62254) are presented in Fig. 4A1, A2, respectively. A total of twelve hallmark gene sets were found to be co-enriched in all two datasets, and the specific details can be found in Table 2. Our analysis revealed that several of two gene sets were associated with cancer and inflammation, indicating their activation in GC patients with elevated levels of CTSK expression. These gene sets include EMT, KRAS signaling up, TNFα signaling via KFκB, angiogenesis, inflammatory reaction, IL2-STAT5 signaling, and IL6-JAK-STAT3 signaling (Fig. 4B1, B2). To a certain extent, the findings of this study indicated that CTSK plays a significant role not only in the inflammatory response associated with GC, but also in the process of cancerization itself. The upregulation of CTSK has been observed to have a detrimental impact on the prognosis of GC patients, potentially mediated by various biological mechanisms.

**Table 2:** Twelve co-enriched activated hallmark gene sets from GSE26253 and GSE62254 datasets

Descriptions	Size	ES	NES	*Adj. p*	Rank
GSE26253					
HALLMARK_ALLOGRAFT_REJECTION	172	0.32	1.40	*	2450
HALLMARK_ANGIOGENESIS	31	0.55	1.80	*	2649
HALLMARK_APICAL_JUNCTION	172	0.37	1.64	**	1848
HALLMARK_COAGULATION	108	0.42	1.74	**	1532
HALLMARK_EPITHELIAL_MESENCHYMAL_TRANSITION	186	0.49	2.22	**	2283
HALLMARK_IL2_STAT5_SIGNALING	181	0.35	1.54	*	2565
HALLMARK_IL6_JAK_STAT3_SIGNALING	75	0.38	1.48	*	2129
HALLMARK_INFLAMMATORY_RESPONSE	166	0.40	1.76	**	2209
HALLMARK_KRAS_SIGNALING_UP	174	0.32	1.40	*	2362
HALLMARK_MYOGENESIS	159	0.43	1.89	**	3465
HALLMARK_TNFA_SIGNALING_VIA_NFKB	184	0.34	1.51	*	2209
HALLMARK_UV_RESPONSE_DN	135	0.42	1.79	**	2321
GSE62254					
HALLMARK_ALLOGRAFT_REJECTION	198	0.72	2.38	****	2779
HALLMARK_ANGIOGENESIS	35	0.79	2.11	****	1708
HALLMARK_APICAL_JUNCTION	195	0.58	1.90	****	2935
HALLMARK_COAGULATION	137	0.62	1.99	****	1677
HALLMARK_EPITHELIAL_MESENCHYMAL_TRANSITION	199	0.85	2.79	****	1884
HALLMARK_IL2_STAT5_SIGNALING	198	0.60	1.95	****	2779
HALLMARK_IL6_JAK_STAT3_SIGNALING	87	0.67	2.03	****	3350
HALLMARK_INFLAMMATORY_RESPONSE	200	0.69	2.28	****	2762
HALLMARK_KRAS_SIGNALING_UP	198	0.66	2.17	****	2545
HALLMARK_MYOGENESIS	198	0.63	2.08	****	2651
HALLMARK_TNFA_SIGNALING_VIA_NFKB	196	0.67	2.20	****	3323
HALLMARK_UV_RESPONSE_DN	142	0.69	2.20	****	2657

*Size* the number of genes in gene set, *ES* enrichment score, *NES* normalized enrichment score

Adj.*p*, adjust *p* value; *p < 0.05, **p < 0.01, ***p < 0.001, ****p < 0.0001

### Infiltration immune cell types in the TME of GC

**Fig. 5:**
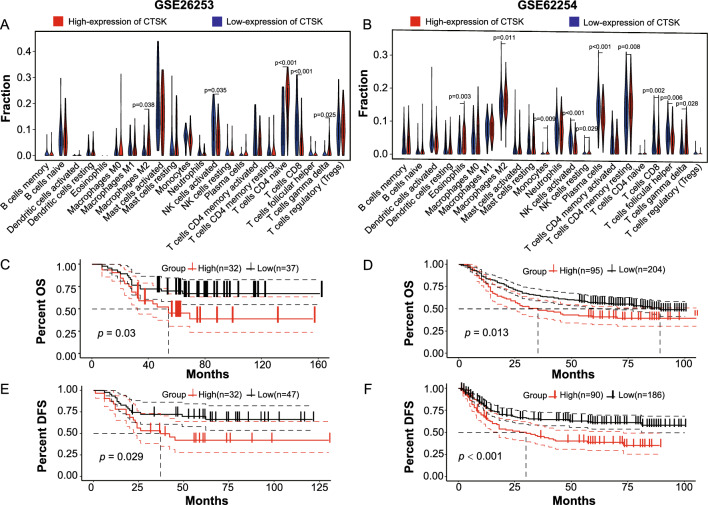
The variations in immune cell infiltration levels between the low and high-CTSK expression groups, and their effects on overall survival (OS) and disease-free survival (DFS) among gastric cancer (GC) patients. **A**, **B** Violin plots for showing the fractions of the 22 immune cell types in GSE26253 (**A**) and GSE62254 (**B**) datasets (blue represents lower-CTSK expression group and red represents higher-CTSK expression group); **C**–**F** survival analysis of M2-macrophages in tumor microenvironment (TME) for GC patients from GSE26253 (OS: **C** and DFS: **E**) and GSE62254 (OS: **D** and DFS: **F**) datasets. *OS* overall survival, *DFS* disease-free survival, *high* GC patients with high-CTSK expression, *low* GC patients with low-CTSK expression. *p < 0.05, **p < 0.01, ***p < 0.001, ****p < 0.0001

Figure 5A, B illustrate the levels of immune cell infiltration between the groups characterized by low and high levels of CTSK expression in the GSE26253 and GSE62254 datasets, respectively. The bar plot illustrating the proportions of the 22 immune cell types from each sample in those two datasets is presented in Figure S2 of Supplementary Material 2. Our analysis revealed that, in comparison to the low-level CTSK group, the high-level CTSK expression group exhibited higher infiltration levels of M2-macrophages and gamma delta T cells, while the level of CD8 T cells was lower in both the GSE26253 and GSE62254 datasets. These findings suggest that GC patients with over-expression of CTSK exhibit high levels of infiltration by M2-macrophages. Survival analysis indicates that M2-macrophages significantly impact OS and DFS rates in GC patients. The results are depicted in Fig. 5C (OS for GSE26253) and Fig. 5E (DFS for GSE26253), and Fig. 5D (OS for GSE62254) and Fig. 5F (DFS for GSE62254). Furthermore, despite the presence of a certain level of CD8T cell infiltration in our findings, the survival analysis results lacked persuasiveness (Figure S3 in Supplementary Material 2). Additionally, variations in gamma delta T cells populations were observed in GC patients with varying levels of CTSK expression; however, due to their minimal proportion, their practical significance in the diagnosis and treatment of GC is limited. Consequently, our study primarily concentrates on assessing the involvement of M2-type macrophages in GC patients with diverse CTSK expression levels. These outcomes suggested a significant association between CTSK and macrophages, implying that CTSK might contribute to the development of immunosuppressive TME in GC patients.

### CTSK promotes the ability of cell proliferation and invasion in GC cells

**Fig. 6:**
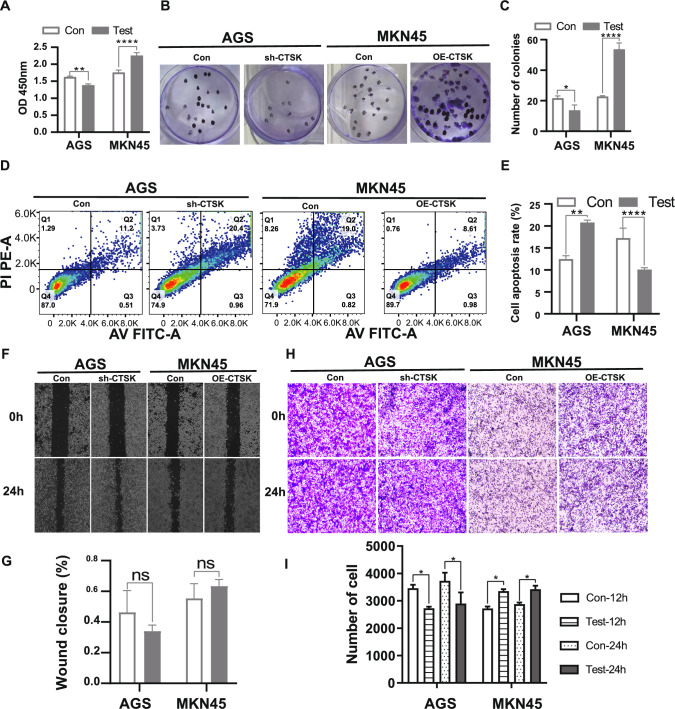
In vitro experiments confirmed the role of different expressed levels of CTSK on GC cells. **A** Bar plot showing the differences of cell proliferation ability between the groups of knockdown or over-expression of CTSK in AGS and MKN45 cells after 48 h of culture. **B** Representative images of the cell ability of colony formation in AGS cells with knockdown of CTSK and in MKN45 cells with CTSK over-expression after 24 h of culture. **C** Comparison of the number of colony formation of AGS and MKN45 cells before and after the intervention. **D** Representative images of cell apoptosis in AGS cells with knockdown of CTSK and in MKN45 cell with CTSK over-expression. **E** Comparison of the total cell apoptosis rate of AGS and MKN45 cells before and after the intervention. **F** Representative images of the healing speed of scratches in AGS cells with knockdown of CTSK and in MKN45 cell with CTSK over-expression. **G** Comparison of the wound closure of AGS and MKN45 cells before and after the intervention. **H** Representative images of the cells invading through a matrigel-coated membrane in AGS cells with knockdown of CTSK and in MKN45 cell with CTSK over-expression. **I** Comparison of the number of cells that invaded through a matrigel membrane in AGS and MKN45 cells before and after the intervention. OD450nm, the value of optical density at 450 nm wavelength. Con, control group, Test in AGS cell lines represents knockdown CTSK group, Test in MKN45 cell represents over-expression CTSK group, sh-CTSK represents knockdown CTSK gene group. OE-CTSK represents over-expressed CTSK gene group. *p < 0.05, **p < 0.01, ***p < 0.001, ****p < 0.0001. *ns* no statistical significance

The cell proliferation capacity of the four candidate cell lines was assessed at 24 h, 48 h, and 72 h, and the comprehensive findings are illustrated in Figure S4 in Supplementary Material 2. Based on the CCK-8 results, the OD450nm value of CCK-8 detection decreased in CTSK knockdown AGS cells compared to the control group, whereas it increased in CTSK overexpression MKN45 cells after 48 h of culture, with statistical significance observed (*p* < 0.05, Fig. 6A); Similarly, the number of colony formations in AGS cells with CTSK knockdown decreased, whereas in MKN45 cells with CTSK over-expression, it increased after 1 week of culture, these results were also statistically significant (*p* < 0.05, Fig. 6B, C); The AGS cells with suppressed expression of the CTSK gene displayed an increase in both early and late apoptotic cells, whereas the MKN45 cells with enhanced expression of the CTSK gene exhibited a decrease (Fig. 6D). The overall rate of apoptosis in each group was measured, revealing a statistically significant rise in the total apoptosis rate of AGS cells with CTSK gene overexpression, and a significant decline in the total apoptosis rate of MKN45 cells with CTSK gene knockdown (*p* < 0.05, Fig. 6E). Cell migration and invasion capability was assessed using wound healing and transwell assays. The results indicated that the percentage of wound closure decreased in AGS cells with knockdown of CTSK, while it increased in MKN45 cells with CTSK over-expression, compared to the control group. However, these differences were not statistically significant (*p* > 0.05, Fig. 6F, G). Additionally, we quantified the number of cells invading through a matrigel-coated membrane in AGS cells with CTSK knockdown, MKN45 cells with CTSK over-expression, and their respective control groups after 12 and 24 h of culture. The findings revealed a decrease in the number of invading cells in AGS cells with CTSK knockdown, while an increase was observed in MKN45 cells with CTSK over-expression, as compared to the control group (Fig. 6H). Although the differences were not substantial, they were statistically significant among the groups (*p* < 0.05, Fig. 6I). These findings suggest that CTSK enhances the proliferation and invasion capabilities of GC cells, although additional assessment is needed to determine the impact of CTSK on GC cell migration.

## Discussion

This study primarily aimed to assess the impact of CTSK expression level on the occurrence and progression of GC. By conducting an extensive analysis of gene expression profiles from multiple databases and performing IHC on CTSK protein in GC tissue specimens, we observed a significant upregulation of CTSK in tumor tissues. This finding suggests a potential role of CTSK in promoting oncogenesis within the human stomach. Our study revealed that GC patients exhibiting a heightened level of CTSK expression not only displayed unfavorable tumor characteristics, but also exhibited the activation of hallmark gene sets associated with various biological processes. These processes include angiogenesis (characterized by the up-regulation of genes involved in blood vessel formation), EMT (defined by genes involved in EMT), inflammatory response (defined by genes involved in inflammatory processes), KRAS signaling up (genes up-regulated upon KRAS activation), TNFα signaling via NFκB (genes regulated by NF-κB in response to TNF), IL2-STAT5 signaling (genes up-regulated by STAT5 in response to IL2 stimulation), and IL6-JAK-STAT3 signaling (genes up-regulated by IL6 via STAT3). The aforementioned evidence suggests that CTSK plays a significant biological role in the development of GC, contingent upon gene sets with specific biological functions. Additionally, our assessment of immune cell types revealed heightened levels of M2-macrophages, CD8 T cells, and gamma delta T cells in GC patients with elevated CTSK expression. Furthermore, our survival analysis demonstrated a noteworthy association between the infiltration level of M2-macrophages and the rates of OS and DFS in GC patients. Moreover, the in vitro evidence strongly indicates that over-expression of CTSK significantly enhances the proliferative and invasive capacities of GC cells, while having no discernible impact on their migratory ability. However, our analysis of CCK-8 results revealed that there was no statistically significant difference observed in either CTSK knockout AGS cells or CTSK overexpression MKN45 cells at 24 and 72 h. The absence of statistical significance at 24 h may be attributed to the selection of the early stage of cell growth for the experiment, while the lack of statistical difference at 72 h may be attributed to the attainment of maximum cell growth. Consequently, our findings offer a novel perspective on the potential involvement of CTSK in tumor progression and immunology, thereby highlighting its viability as a prospective target for curtailing the invasion of GC cells.

One of the primary distinctions observed between the high-level and low-level groups of GC patients is the activation of hallmark gene sets associated with carcinogenesis and cancer-related inflammation in the high CTSK expression group. Our findings suggest that CTSK plays a crucial role in the biological mechanism underlying the induction of an inflammatory response and EMT. It is widely acknowledged that EMT and inflammation synergistically contribute to the development of GC [26–29], with a robust association between EMT and inflammation in human physiology [30, 31]. Moreover, it is worth noting that the process of EMT is a ubiquitous characteristic observed in epithelial cancers, and it is closely associated with the aggressive nature of tumors and their ability to metastasize [32, 33]. In our investigation, we have discovered that the expression of CTSK is not only upregulated in GC tissues compared to normal tissues, but it is also significantly overexpressed in high-stage tumors (TNM staging: III, IV, and T-staging: T3, T4) when compared to low-stage tumors (TNM staging: I, and T-staging: T1). Besides, it is imperative to note that CTSK exhibits not only significant upregulation in the EMT molecular subtype of GC, but also in diffuse-type GC, which is characterized by a more aggressive behavior and is associated with a poorer prognosis for patients. In conjunction with the findings from in vitro experiments and bioinformatics analysis, obtained evidence suggest a close association between the CTSK gene and the biological behavior of locally invasive growth during the tumorigenesis and progression of GC.

On other hand, tumor related inflammatory response is worthy of interest. The involvement of the STAT (signal transducer and activator of transcription) family assumes a crucial function in discerning whether the immune responses within the TME facilitate or impede cancer progression. Particularly noteworthy is the persistent activation of STAT3 and STAT5, which not only bolster the survival and invasive potential of tumor cells but also expedite their proliferation while concurrently suppressing the anti-tumor immune response. Moreover, previous studies have demonstrated that STAT3 is involved in the regulation of tumor-promoting inflammation [34–36]. The signaling pathways IL2-STAT5 and IL6-JAK-STAT3, which were identified in our findings, are of significant importance in the context of inflammation-associated carcinogenesis [37, 38]. Furthermore, the IL6-JAK-STAT3 pathway has emerged as a promising therapeutic target within the realm of cancer therapy [39–41]. Previous studies have amassed evidence regarding the involvement of the KRAS signaling in various aspects of GC pathogenesis, including the induction of chronic inflammation, facilitation of dysplasia development, guidance for GC treatment, and augmentation of intratumor morphological heterogeneity [42, 43]. Additionally, a recent study has reported a correlation between KRAS activation and EMT, proposing that KRAS may stimulate EMT and foster the generation of cancer stem-like cells (CSCs), thereby promoting metastasis in GC [44]. Tumorigenesis is an outcome of oncogenic mutations, both directly and indirectly, and GC is not exempt from this multifactorial and multistep process. Our study revealed a significant activation of genes associated with carcinogenesis and inflammation in GC patients exhibiting high levels of CTSK expression. These findings provide a potential explanation for the role of CTSK over-expression in promoting GC progression. However, it is imperative to replicate these conclusions and validate the data to ensure their reliability.

Significantly, our observations have yielded intriguing findings regarding the infiltration of immune cells in the TME. Within the human body, macrophages can be categorized into two commonly recognized types: firstly, the M1-phenotype, referred to as classical macrophages, exhibit robust anti-germ and anti-tumor properties [45, 46]; secondly, the M2-phenotype, known as alternatively activated macrophages, play a role in tissue remodeling, angiogenesis, as well as tumor formation and progression [47, 48]. In general, tumor-associated macrophages (TAMs) serve as crucial regulators of the tumor immune microenvironment, resembling M2-like phenotypes, and exert immunosuppressive effects, which have garnered significant attention in current research endeavors [47, 49–51]. Multiple studies have documented a strong correlation between the infiltration levels of macrophages and the advancement of tumors [52–54]. Our study in GC patients has substantiated the significant association between M2-macrophages and inferior OS and DFS rates. Moreover, numerous studies have consistently reported that an elevated level of M2-macrophage infiltration is linked to peritoneal dissemination, angiogenesis, immune evasion, and an unfavorable prognosis [55–59]. The ectopic expression of genes within tumor tissues has the potential to stimulate the recruitment of immune cells into the TME, either directly or indirectly, through the action of inflammatory mediators released by GC cells or infiltrating cells [60–63]. The findings of our study indicated that GC patients exhibiting elevated CTSK expression are characterized by notable activation of an inflammatory gene sets and heightened infiltration of M2 macrophages. In other words, these observations indicated a significant association between the expression level of CTSK in patients with GC and the extent of inflammation surrounding tumor cells, as well as the infiltration of M2 macrophages. The up-regulation of CTSK by GC cells had the potential to enhance the advancement of tumor-associated inflammatory mechanisms within the TME, as well as attract supplementary M2 macrophages to the proximity of tumor cells, thereby potentially fostering the development of an immunosuppressive microenvironment.

**Fig. 7:**
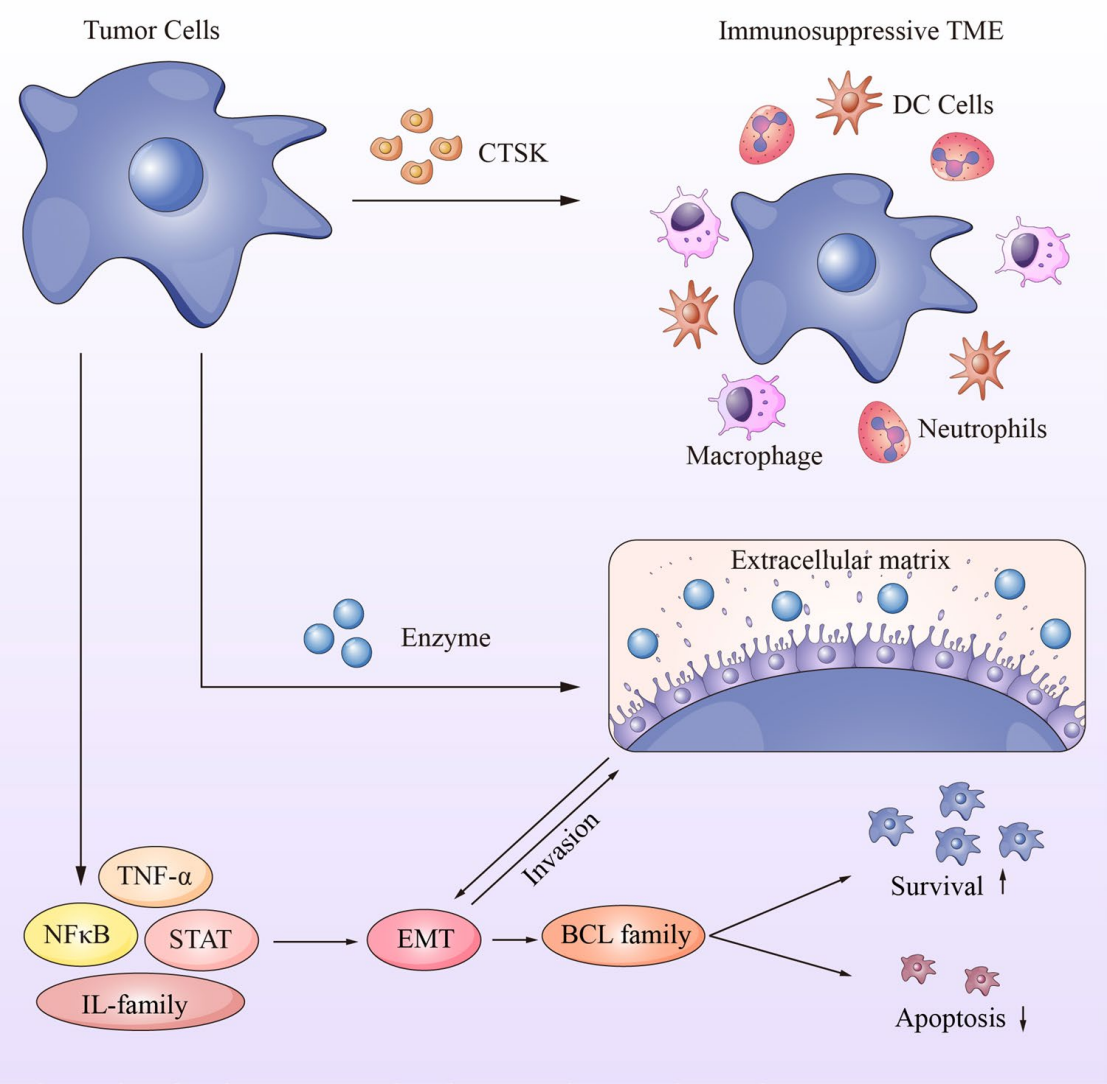
Graph of the potential mechanism of CTSK involving in GC progression. *TME* tumor microenvironment, *DC cells* dendritic cells, *TNFα* tumor necrosis factor, *NFκB* nuclear factor kappa-B, *STAT* signal transducer and activator of transcription, *IL-family* interleukin family, *EMT* epithelial–mesenchymal transition, *BCL-family* B-cell lymphoma family

CTSK, a gene associated with the extracellular matrix (ECM), has the ability to induce alterations in stromal structure and facilitate the degradation of ECM. Consequently, this process leads to the remodeling of ECM, a crucial factor in the initiation and advancement of EMT [64, 65]. Thus, it can be inferred that an association exists between CTSK and its abnormal expression, and the occurrence of EMT (Fig. 7). As previously discussed, immune cells associated with tumors possess the capacity to either eliminate tumor cells or facilitate their progression and metastasis. Regrettably, in the majority of cases, immune cells within the TME tend to collaborate with tumors rather than combat them. The findings from our survival analysis support the notion that immune cells play a crucial role in GC. Specifically, the over-expression of CTSK led to substantial alterations in the immune cell composition within the TME of GC patients. Notably, there was a decrease in immunocytes with cytotoxic capabilities, while immunocytes with inhibitory functions exhibited an increase. These changes fostered the development of immunosuppressive microenvironments that favor tumor cell survival (Fig. 7). In conclusion, our hypothesis is that elevated expression of CTSK may impact the advancement of GC by enhancing the invasive capacity of GC cells and stimulating the inflammatory response in the vicinity of tumor cells. To validate this conjecture, we constructed CTSK knockdown and overexpression cell models in GC cell lines and conducted in vitro experiments to assess the effects of varying levels of CTSK expression on GC cells. The ultimate findings substantiated that CTSK overexpression indeed facilitated the proliferation and invasion of GC. Nevertheless, this study is subject to certain inherent limitations. Firstly, the impact of CTSK overexpression on the migratory capacity of GC cells lacks corroborating experimental evidence. Secondly, the absence of direct evidence linking CTSK to EMT is noteworthy. Thirdly, the precise interconnections among CTSK, EMT, inflammatory response, and the signaling pathways involving IL and STAT family members necessitate prospective validation (Fig. 7).

## Conclusion

CTSK has the potential to facilitate the initiation and progression of GC by augmenting the invasive capacity of GC cells, engaging in tumor-associated EMT, and fostering the establishment of an immunosuppressive TME. Consequently, CTSK assumes significance in the context of tumor immune evasion and emerges as a promising candidate for impeding the invasion of GC cells.

### Supplementary Information

Below is the link to the electronic supplementary material.

Supplementary file 1 Details of data pre-process and experiment procedures and genes of core enrichment in the 12 co-enriched hallmark gene sets in two cohorts from GEO database. (DOCX 505 KB)

Supplementary file 2 (DOCX 1570 KB)

Supplementary file 3 (DOCX 32 KB)

Supplementary file 4 (XLSX 18 KB)

## Data Availability

The original contributions presented in the study are included in the article/supplementary material. Further inquiries can be directed to the corresponding author.
