# Supplementary material for: The potential contribution of aberrant cathepsin K expression to gastric cancer pathogenesis
Source: Discov Oncol. 2024 Jun 10;15:218. doi: 10.1007/s12672-023-00814-z (PMC11164852; doi:10.1007/s12672-023-00814-z)
Supplement: Supplementary file 1 — Details of data pre-process and experiment procedures and genes of core enrichment in the 12 co-enriched hallmark gene sets in two cohorts from GEO database. (DOCX 505 KB) [file 12672_2023_814_MOESM1_ESM.docx]

Supplementary Material 1

# Details of datasets selection

Datasets were retrieved from GEO database (https://www.ncbi.nlm.nih.gov/geo/) with “gastric cancer” as the key word. The filters of organism and study type were limited as “Homo sapiens” and “Expression profiling by array”, respectively. Then the gene expression matrix and the corresponding platform TXT files of target datasets were downloaded. R software (R 3.6.1, https://cran.r-project.org/) and related packages (http://www.bioconductor.org/) were used for data processing. The datasets utilized to differential analysis must be satisfied the following conditions: (I) all of the sequencing samples were from the patients with GC or gastric tissues in Asian population; (II) the datasets for differential gene analysis contained controls for cancer and canceradjacent tissues or non-tumor gastric tissues; (III) the sample size of each dataset was at least 50; (IV) the information of the platform annotation was available.

Table s1 Details of datasets used in the study from the GEO database. N_tum_,sample number of gastric cancer; N_nor_, sample number of normal gastric tissues; ACRG, Asian Cancer Research Group.

| **GEO _id_** | **Country** | **N_tum_** | **N_nor_** | **GPL _id_** | **Explain** |
| --- | --- | --- | --- | --- | --- |
| GSE2669 | Singapore | 58 | 64 | GPL2048 | Exploring expression levels: removed data with only one sample: GSM51580 and GSM51585. |
| GSE54129 | China | 111 | 21 | GPL570 | Exploring expression levels: all samples included in the analysis |
| GSE65801 | China | 32 | 32 | GPL14550 | Exploring expression levels: all samples included in the analysis |
| GSE84437 | South Korea | 432 | 0 | GPL8432 | Exploring expression levels: all samples included in the analysis |
| GSE62254 | ACRG Cohort | 300 | 0 | GPL570 | Survival analysis and GSEA cohort |
| GSE26253 | South Korea | 432 | 0 | GPL8432 | Survival analysis and GSEA cohort |

After searching and filtering, we used R software (Version R 3.6.1, https://cran.r-project.Org/) to finish subsequent analysis. The “GEOquery” package was used to download the gene expression matrix and corresponding annotation platform files of the target datasets, and then extracted the gene expression matrix using the “exprs” function. The clinical features of samples matching the expression matrix were extracted using the “pData” function. We used the “limma” package in R software to standardize the candidate datasets for differential analysis, and converted the gene expression matrix to log2. After completing the above steps, gene differential expression analysis was performed again between GC tissue samples and adjacent (or normal) tissue samples using the Limma package. When a gene simultaneously satisfies | log fold change (FC) |>1 and adjust *p*<0.05, it is considered as a differentially expressed genes (DEGs).

Pca analysis:


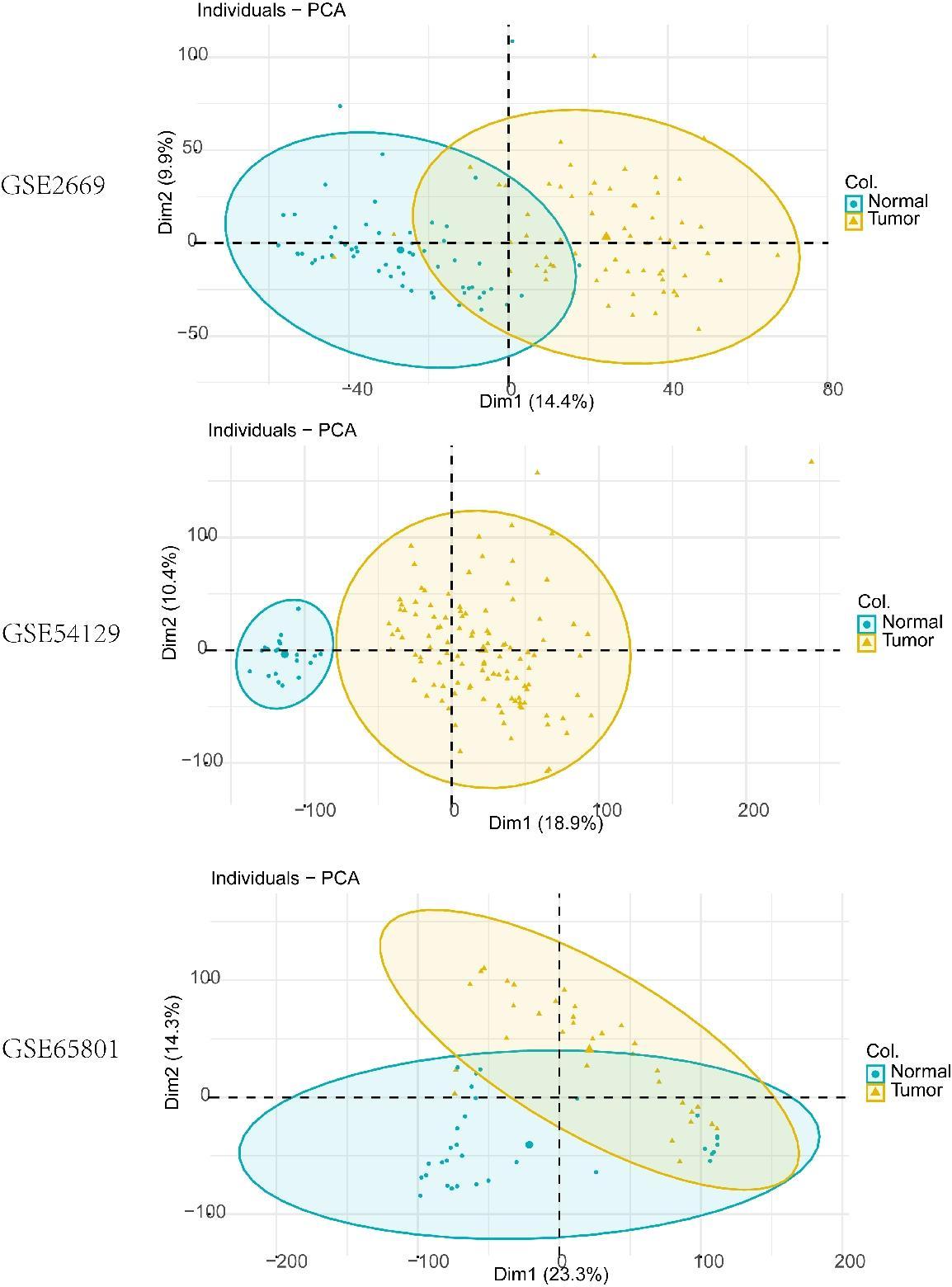
Figure s1 The results of principle component analysis for GSE2669,GSE54129, and GSE65081 datasets.

# Details of experiment procedures

## IHC staining

1. Main experimental reagents:

① Antibody diluent: PBS 100ml, BSA 0.2g, sodium azide 0.03g, Triton 100ug.

② 0.1% trypsin: trypsin 0.1g, 0.1% calcium chloride (pH 7.8) 100ml.

③ 0.3% methanol-H2O2 solution: 100ml pure methanol, 1ml H2O2.

④ 0.01mol/L PBS buffer: Na2HPO4· 12H2O 14g, Na2HPO4· 12H2O 1.5g, NaCl 45g, distilled water 5L. After the salt was fully dissolved, the pH was adjusted to 7.2-7.4 with NaOH.

⑤ Citrate buffer: first, 21.01g citric acid was added to 1L distilled water to make 0.1mol/L citric acid solution, and 29.41g sodium citrate was added to 1L distilled water to make 0.1mol/L sodium citrate solution. However, the concentration of citrate buffer used for microwave antigen repair is 0.01mol/L, so it needs to be temporarily used. The concentration is as follows: take 9ml 0.1mol/L citric acid solution and 41ml 0.1mol/L sodium citrate solution, add 450ml distilled water after mixing, and maintain the pH range of 6.0.

⑥ DAB color solution: DAB (3, 3-diaminobenzidine tetrahydrochloride) 50mg, PBS solution 100ml, 30% H2O2 (current preparation) 30-40ul. DAB should be dissolved with a small amount of PBS in advance, and the remaining PBS can be added after the DAB is fully dissolved. Attention should be paid to avoid light during operation. The mixture of DAB and PBS should be shaken and filtered, and 30%H2O2 should be added before color development.

3. Operation steps:

① Baked slices.

② Deparaffinized to water: After being placed in xylene Ⅰ, Ⅱ and Ⅲ for 10min, the tissue sections were immersed in absolute ethanol, 95% ethanol, 80% ethanol and 75% ethanol for 3min, respectively.

③ Rinse with running water: rinse off the ethanol on the section.

④ Antigen repair: high pressure heat repair, add appropriate pH 6.0 citrate buffer into the pressure cooker, after venting for 3 minutes and cooling for 20 minutes.

⑤ 3%H2O2 was kept in the dark for 15min (30%H2O2: deionized water =1:9), and washed with PBS for 3 times.

⑥ The slides were added with appropriate amount of primary antibody and incubated at 4℃ overnight.

⑦ PBS washing; Rabbit secondary antibodies were added dropfold and incubated at room temperature.

⑧ DAB color: wash 3 times with PBS, 5min each time (shaking slowly); DAB color solution (A:B=1:50) was prepared, 100ul of color solution was used, and incubated for 3-5min. When obvious brown-yellow immunoblot was observed under the microscope, staining was terminated with PBS.

⑨ After hematoxylin staining, the cells were rinsed with running water and differentiated with ethanol hydrochloride. After dehydration with gradient ethanol to wax, xylene Ⅰ and Ⅱ for 5min each, the slices were sealed with neutral gum after drying.

4. Result Judgment:

The location and depth of IHC staining reflect the location and amount of antigen, which can be used as a basis for qualitative, localization and quantification [103]. The staining of positive cells can be focal or diffuse, and can be in the cytoplasm, nucleus or cell membrane surface. The staining intensity of different cells can be different, and the judgment of the results needs to set a strict control. Therefore, normal tissues matched to GC tissues were used as controls in this study for judgment analysis of the results. In addition, to ensure the reliability of IHC staining results, specific staining and non-specific staining were also differentiated during the analysis of the results. The judgment of the final score of IHC staining was detailed as follows : the score of staining intensity was as follows: 0, negative; 1, weak coloring; 2, moderate staining; 3. Strong coloring. The score corresponding to the proportion of positive cells was defined as follows: 0, &lt; 5%; 1, 5-25%; 2, 26%-50%; 3, 51%-75%; 4, more than 75%. The final result was determined by multiplying the staining intensity score by the positive area (proportion of positive cells) score to determine the staining index (index range: 0-12), with a score of 0-7 being considered low expression and a score of 8-12 being considered high expression.

## Processing procedures of public databases

The main processes of data download, standardization, data extraction and analysis were based on R software: For STAD data, we used the download tool of TCGA database, and then used the DESeq2 package in R software [105] to standardize the data. After that, the CTSK expression matrix was constructed and extracted, and the clinical data of GC patients in TCGA database were extracted at the same time, including GC infiltration stage (T), GC infiltration stage (T), and GC infiltration stage (T). Lymph node status (N) and distant metastasis (M) were integrated with CTSK expression matrix to obtain CTSK expression matrix with clinical features. For the candidate data sets in the GEO database, the GEOquery package was used to download, and the exprs and pdata functions were used to extract the gene expression matrix and the matching sample information, respectively. The gene expression matrix was standardized by the limma package [23] and log2 transformed. Finally, the data were integrated with the matched clinical data of the samples. Finally, statistical analysis of the data was performed using GraphPad Prism (Version 8.0) and R software (see Statistical Analysis for details). The results were presented in the form of box and dot plot combination, and *p* < 0.05 was considered as statistically significant.

## Cell line culture

1. Selection of human GC cell lines

The human GC cell lines AGS, HGC-27, MKN-45, NCI-87, and the human gastric mucosal normal cell line GES-1 were purchased from the ATCC cell repository.

1. Main reagents and consumables
2. well plate, 24-well plate, 96-well plate, 25cm2 Cell culture flask, 75cm2 Cell culture dishes are the products of Corning (USA) , PCR eight-connected pipe set and EP pipe are products of Axygen Company; The gun head is from American KIRGEN company; For cell culture, combined antibiotics (penicillin, streptomycin, meil, vancomycin) for Aladdin products; PBS buffer (pH 7.2-7.4), HRP labeled goat anti-mouse / rabbit antibody, difluorinated resin (PVDF) fiber membrane are the products of Beijing Zhongshan Jinqiao Company; RIPA protease inhibitors, SOS-PAGE gel preparation kit, BCA protein quantification kit and so on are the products of Verde Biotechnology Co., LTD.; Fetal calf serum, RMPI1640 medium, DMEM medium, and trypsin containing 0.25% EDTA are all products of Gbicico; The above reagents and consumables are purchased by Lanzhou Lianchuang Biotechnology Co., Ltd. from the relevant manufacturers.
3. Cell resuscitation

Under the guidance of the experimental methods listed in the refined Molecular Biology Experimental Technology, the related operations of cell resuscitation:

Adjust the temperature of the water bath to 36℃ constant temperature and preheat the cell complete medium; at the same time, Prior UV irradiation for 15min, wear gloves, Alcohol to disinfect hands and spray ultra-clean countertops; Place 1ml of cell complete medium in 15ml, centrifuge tube; Adjust the water bath temperature to a constant temperature of 40℃, Crystold cells were removed from the liquid nitrogen tank, Quickly place the freezer tube in a water bath that has been preheated to 40℃, And kept shaking, Quickly thaw the crystock liquid in the tubes, During the operation, attention should be paid that the cell freezing mouth must be higher than the water surface, To reduce the chance of the cells being contaminated. After 1min of thawing, the outer wall of the cell tube was sprayed with 75% alcohol and sent to the ultra-clean table for cell suspension preparation.

1. Prepared cell suspensions

The liquid in the freezer was placed in a 15ml centrifuge tube already in the cell complete medium with a pipette, mixed up and down, centrifuged at 1000rpm at room temperature for 5min, and the supernatant was discarded.

1. The cell culture

The pipette absorbed 1ml cell complete medium into 15ml centrifuge tube and was gently blown to make cell suspension; 25cm2Add 4ml of complete medium to the bottle, add 1ml of cell suspension to the bottle, gently shake the cells; add 37℃ with 5%CO2 cell culture incubator culture.

1. Results of the observation

After 24 hours, cell adhesion growth can be observed. According to the state of cell growth, 1~2d is replaced with new medium; besides, pay attention to the abnormal appearance of cell growth such as no pollution and death.

## mRNA extraction and quantification

1. Main experimental instruments and reagents

UV spectrophotometer, TRIzol RNA separation reagent (The rmo Fisher products, purchased by Lanzhou Lianchuang Biotechnology Co., LTD.), chloroform (trichloromethane), isopropanol, ethanol, DEPC treated water, etc.

1. Operation steps

① TRIzol Processing

Cell culture medium was aspirated from the pipette, cells were washed twice using PBS, Trizol added and lysed at room temperature. The cells were evenly blown down by the pipette and transferred to the EP tube, gently reversed up and down, and let sit at room temperature for 5min.

② Add 1 / 5 volume of chloroform (e. g. 1ml Trizol plus 0.2ml chloroform), mix well 10 times, stand at room temperature for 5min, 4℃, 12000 r/min, centrifugation for 20min.

③ Turn the upper aqueous phase into a new EP tube, add equal volume of isopropanol, mix well, stand at room temperature for 10min, 4℃, 12000 r/min, centrifugation for 15min.

④ The supernatant was carefully aspirated with a pipette, and a precooled 75% ethanol was added, 4℃, 12000 r/min, centrifuged for 10min, and the supernatant was discarded.

⑤ EP tube was inverted and air dried for 5min.

⑥ EP tubes were added with DEPC treatment water to make a solution containing total cellular m RNA.

⑦ The mRNA dissolved in DEPC-treated water was appropriately diluted and then quantified using a UV spectrophotometer.

Note that after starting the quantitative instrument, use 100ul deionized water to make the blank reading to remove the background, and then test the sample.fetch OD260Value and OD260/OD280specific value.

1. Results of the interpretation

Confirm the ratio of OD260/OD280. If the ratio is between 1.8 and 2.0, the next step can be reversed. Final mRNA concentration = OD260Values x 40 x dilution / 1000.

## Protein extraction

1. Main experimental instruments and reagents

EP tube, PBS buffer, phenylmethyl sulfonyl fluoride (P MSF) (aladdin company product, by Lanzhou Lianchuang Biotechnology Co., LTD.).

1. Operation steps:

① When the cells were cultured to about 80% density, the cell medium was discarded and rinsed with precooled PBS buffer (0.01M pH 7.2~7) to remove the residual medium. Finally, the pipette aspirated the PBS buffer and placed the culture bottle on ice.

② The lysate was prepared at the ratio of 1ml of lysate plus 10ul of phenylmethylsulfonyl fluoride (P MSF), shaken well and placed on ice.

③ Add 200ul of lysate containing P MSF to each cell on ice for 30min. For sufficient cell lysis, the lysate was added evenly and the culture bottle was gently shaken.

④ After the lysis process, the cells were scraped on one side of the culture flask, and then the contents were transferred to a 1ml EP tube.5

⑤ EP tube was centrifuged at 12000rpm for 5min.

⑥ The supernatant was total cellular protein, which was moved into a 0ml centrifuge tube and temporarily stored in-20℃ refrigerator..5

## Protein quantification

Quantification of the extracted proteins from each cell line was performed using the BCA (bicinchonininc acid) method.

1. Main experimental reagents and consumables:

Protein samples, BCA Protein quantification kit (Abcam product, held by digestive tumor laboratory, containing BCA Reagent A, stored at room temperature; Cu Reagent B, room temperature; BCA standard 4 mg/ml, -20℃ frozen), EP tube, 96-well plate, microplate reader.

1. Operation steps:

① Configure the working fluid:

Arrange 50 volume BCA reagent plus 1 volume Cu reagent into BCA working solution (all included in the kit) and mix well.

② Standard BSA with double steam, 0.9% saline, PBS or buffer of protein sample to be tested: 100ul 4000u g/ml BSA + 100 ul dilution solution = 200ul (BSA = 2000 ug / ml), 100ul serial dilutions 7 times, BSA standard solution 2000ug/ml, 1000ug/ml, 500ug/ml, 250ug/ml, 125ug/ml, 62.5ug/ml, 31.25ug/ml, 15.625ug/ml.

③ Add 200 ul of AB mixture to each 96-well plate, add 25ul of standard and sample to the mixture, and mix well.

④ Was placed in an incubator, incubated at 37℃ for 30min, and then let to stand at room temperature for 10min.

⑤ The absorbance was detected at 562nm and the sample protein concentration was calculated according to Eq. As in the measured sample OD562=0.8 (i. e., y=0.8), then the corresponding X value (sample protein concentration) = = (0.8-0.1214)/0.0003=2262ug/ml (i. e., 2.3 ug / ul).

⑥ Adjust the total protein amount according to the protein concentration, and 1 / 5 volume of 5X loading buffer was added to cook at 95℃ for 5min, which could be loaded or placed in 4℃.

## Real-time quantitative (Real Time Quantitative, RT-Q) PCR

1. CTSK, and primer synthesis

Through the National Center for Biotechnology Information (National Center for Biotechnology Information, NCBI) Database and Ensembl, the database queries the transcriptomic sequence about CTSK mRNA, The commonly used transcripts were identified as CTSK-201 (Transcript ID: ENST00000271651), Its corresponding base pair length is 1629bp, The sequence was then submitted to BGI for further primer design and synthesis (Contract No. NO: C QP10272050883), The final CTSK primers are as follows: Primer Name (Sequence (5´ to 3´): TTCCAGTTTTACAGCAAAGGTG, GC%:40%); Reverse Name (Sequence(5´ to 3´):CTGGTTTCTTATTTCGAGCCAT, GC%:40%).

1. For cDNA synthesis

The reverse transcription kit (EvoM-MLV is a bioproduct, purchased by Lanzhou Lianchuang Biotechnology Co., Ltd.) is performed in two steps: the first step is to remove genomic DNA, and the second step is to reverse transcription reaction. G APDH As a control, the relative gene expression levels were determined using 2-△△The Ct method was calculated. PCR were performed according to PCR Reaction Kit (Takara) instruction.

## Western-Blot experiment

1. SDS-PAGE:

Main reagents and instruments:

Pre-stained protein marker, electrophoresis buffer, and protein electrophoresis apparatus

1. Operating steps:

⑴ Clean the glass plate

⑵ Guhesing and loading

① Align glass plate, clamp after vertical clamp on the shelf, ready to glue. Before filling the glue, fill the water between the glass plates to check the leakage.

② Select the corresponding concentration of separation glue according to the molecular size of the target protein (Table 2.5-6, configuration (Table 2) refer to the details listed in the Precision Molecular Biology Experimental Technology [102]). When configthe gel, the premise of full mixing. After the completion of the gel perfusion, a layer of water is added to squeeze the gel on the one hand and accelerate the gel on the other hand. SDS-PAGE separation glue concentration and optimal separation range were performed according to instruction.

③ When there is a clear refractive line between the water and the glue, the gelatin has solidified. At this point, pour the water from the top glue and absorb the paper for the remaining water in the glass board.

④ Arrange the concentrated glue according to the instructions (Table 2.7: P 115-116 [102]), shake well and fill the glue immediately. After filling the remaining space with concentrated gel, insert the comb into the comb level; remove the comb before electrophoresis.

3. Cataphoresis

① Mix the appropriate volume of samples for 6x L oading buffer and heat at 100℃ for 5min.

② After the configured glue is removed from the clip, mounted vertically on the electrophoresis tank together with the glass plate, check the tightness of the rubber plate and the electrode frame, and fill the inner pool with electrophoretic buffer (formula as shown in Table 2.8). If there is no significant change in the liquid level within 5min, the gelatin plate is close to the electrode frame and there is no leakage, which can be used for experiments. Cover the adhesive plate 2-3cm in the outer pool.

③ Draw the sample with the pipette attachment, insert the gun head into the sample hole, slowly add the sample, and add the sample and protein predye marker according to law. The maximum loading volume of 15 well 1mm glue and 30ul of 10 well 1mm glue..5.5

④ After the loading is completed, turn on the power supply and conduct electrophoresis in the shortest time to avoid the diffusion of protein bands and affect the results. Electrophoretic time and voltage were determined and adjusted according to the practice of each laboratory and the size of the target protein. Electrophoresis can be stopped until bromophenol blue just runs out. Under normal circumstances, it can end within 2h and turn the membrane immediately after the electrophoresis. 5% SDS-PAGE concentrated glue configuration and formulation of electrophoretic buffer were performed according to instruction.

4. Electrotransfer

Electrophoretic transfer operation was performed using the semi-dry transfer method.

operating steps:

① After the electrophoresis, remove the rubber plate, and prepare the items needed for electrotransfer: 6 filter paper, 1 P VDF film (soaked with 100% methanol for 20min), the size of the gel, pre-cooled electrotransfer tank and electrotransfer liquid.

② After prying open the glass plate, peel the glue, immediately put it into the petri dish with transfer liquid, and then gently scrape the concentrated gel. In this operation, pay attention to not scraping the separation glue. The cut glue, P VDF film soaked in methanol and filter paper were equilibrated in the transfer solution for 10min.

③ Sandwich operation: place the pre-soaked sponge with transfer liquid, 3 layers of filter paper, gel, nitrate cellulose film, 3 layers of filter paper, sponge in the transfer clip in order, to ensure that there is no bubble between each layer.

Assembly sequence: turn film clip black face (negative electrode) -sponge pad-filter paper-glue-membrane-filter paper-sponge pad-red face (positive electrode).

④ The transfer clip was inserted into the electric transfer tank, and 4℃ of pre-cooled membrane transfer buffer (formula as shown in Table 2.9) was added to connect the gel surface to the negative electrode, and the nitrocellulose membrane to the positive electrode, namely "black to black". Also insert into the built-in ice box. About the current and time: the current of the membrane is adjusted between 200-400 mA, the time is based on the size of the target protein, the smaller the molecular weight, the shorter the time, the larger the molecular weight, the time is appropriately extended.

⑤ After the electric transfer, disconnect the power supply and remove the P VDF film. It can be found that the original marker on the glue has now been transferred to the membrane. In order to avoid the membrane drying, the P VDF film should be immediately soaked in the sealing solution (5% skimmed milk) and sealed at room temperature for 1h.

5. Immunolocalization of the enzyme

Operating steps

① Incubation of the primary antibodies:

The primary antibody was diluted with blocking solution (5% skim milk) to the appropriate concentration and incubated with the blocked P VDF membrane for 4℃ overnight or 2h at room temperature. Membranes were washed three times with T BST on a decolorization shaker for 10min each at room temperature.

② Incubation with secondary antibodies: dilute with T BST containing 5% skimmed milk with the corresponding secondary antibodies (generally 1:1000 or even 1:10000) and incubate for 1h at room temperature. Membranes were washed three times with T BST on a decolorization shaker for 10min each at room temperature.

③ In the dark room, take out the film and spread it on the plastic wrap in the dark box to avoid generating bubbles. The two reagents of ECL were mixed in equal volume in the EP tube, and evenly dropped on the protein surface of the P VDF membrane. After 2min of reaction, the excess ECL working solution on the P VDF membrane was dried, and the other side of the plastic film was turned over and covered on it. Remove an X-ray film (cut a corner to indicate the direction) and cover the film (cover the film). Mark the film position straight along the upper edge with a marker. Cover with the dark box and expose.

④ Pour the 1x developing solution and fixing solution into the plastic plate respectively, remove the exposed film from the dark box, and put it into the developing solution for development. If the strip appears, the film can be removed. After washing the tap water, put it into the fixing fluid. After a while, rinse with water and dry. Place the dry film back to the cartridge in the marked position during exposure. According to the position of the prestained marker on the P VDF film, mark the marker on the film with a marker (the position of the marker band on the film strictly corresponds to the prestained marker on the P VDF film).

⑤ The film was scanned or photographed, and the results were quantitatively analyzed using Image J image processing software.

# Results

## RT-qPCR

We validated the basal expression level of the CTSK gene in the candidate GC cell lines via RT-qPCR array, and the results found that the CTSK gene was highly expressed in AGS, N87, and HGC27 GC cell lines(Figure s2). The RT-qPCR experiments were repeated at least three times.


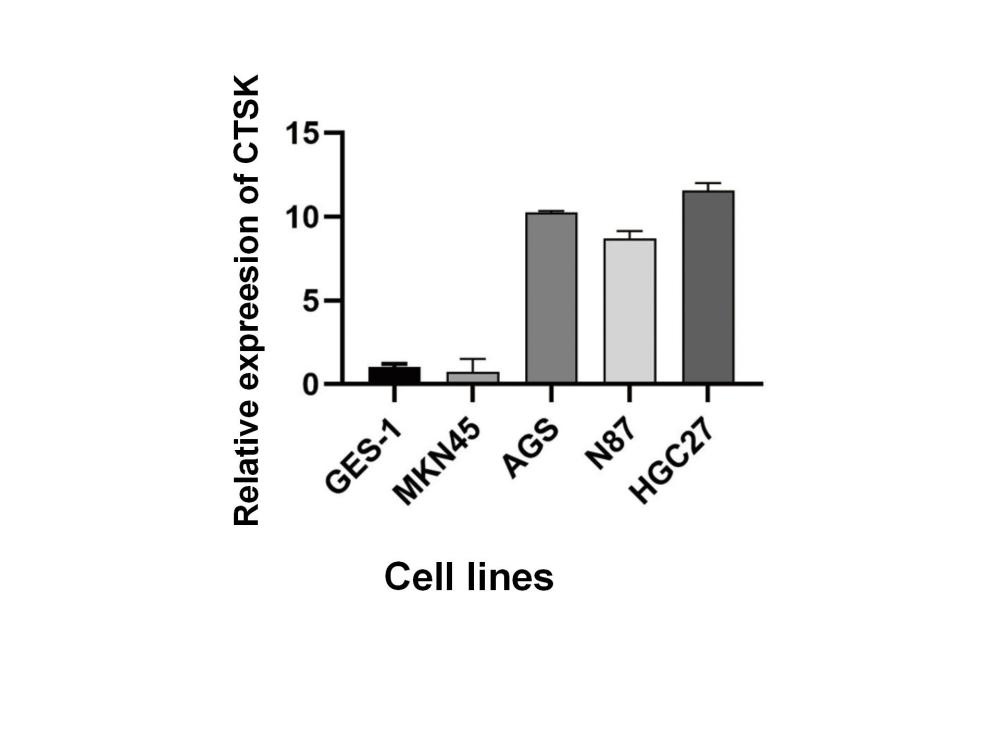
Figure s2 RT-qPCR validated the basal CTSK expression in MKN45, AGS, N87, and HGC27.

As we performed immunohistochemical analysis using the CTSK antibody on patients with GC, and the results had confirmed that CTSK was higher expressed in GC tissues compared with normal tissues. So, here we selected KNM45 cell line as the CTSK lower-expressed research object, and HGC27 cell line as the CTSK higher-expressed one. And then, we used lentiviral vector system for transfection: for MKN45, we over-expressed CTSK gene (Figure s4), and for HGC27, we knockdowned CTSK gene (Figure s4), and both groups established control groups. After transfection, we used RT-qPCR (Figure s5) and WB test (Figure s6) to validate the transfection efficacy, and the results showed that he cells were successfully transfected by lentiviral vectors.


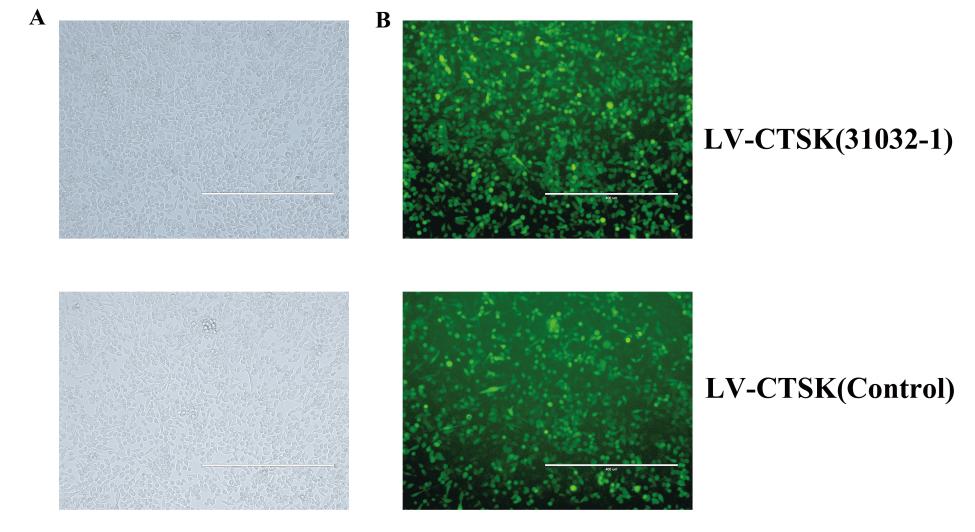
Figure s3 The over-expressed LV transfected MNK45 cell line. Green fluorescence showed the cells that had been successfully transfected with lentivirus


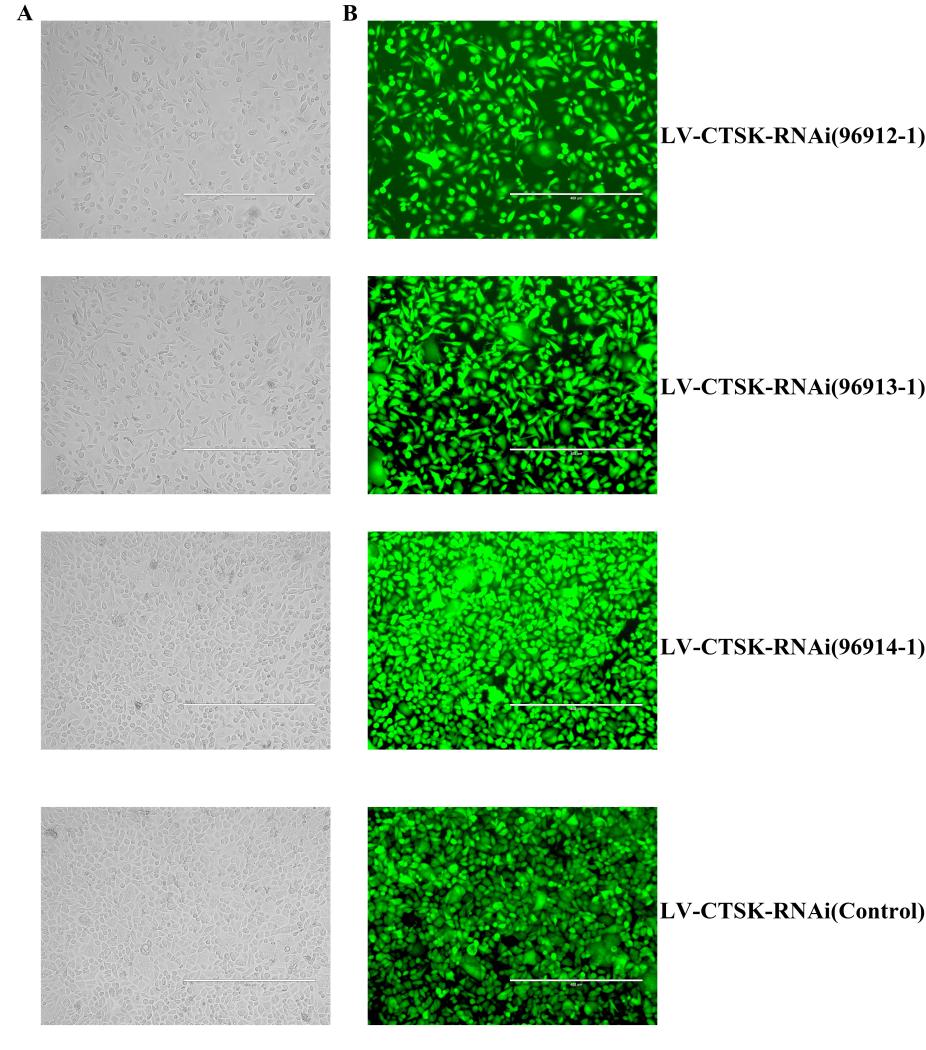
Figure s4 The knockdown-expressed LV transfected AGS cell line. Green fluorescence showed the cells that had been successfully transfected with lentivirus


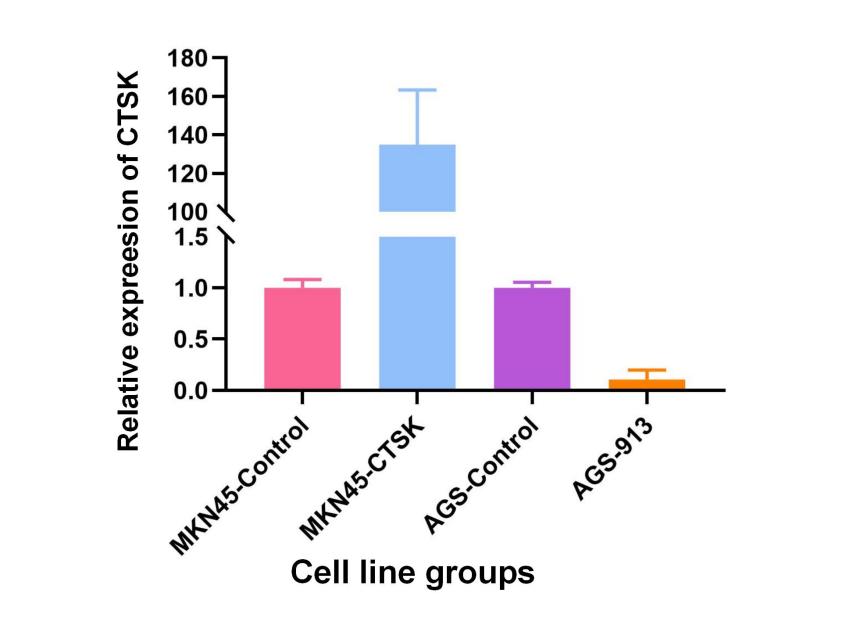


Figure s5 The RTA-qPCR results of oe- and ko-expressed LV transfected MKN45 and AGS cell line.


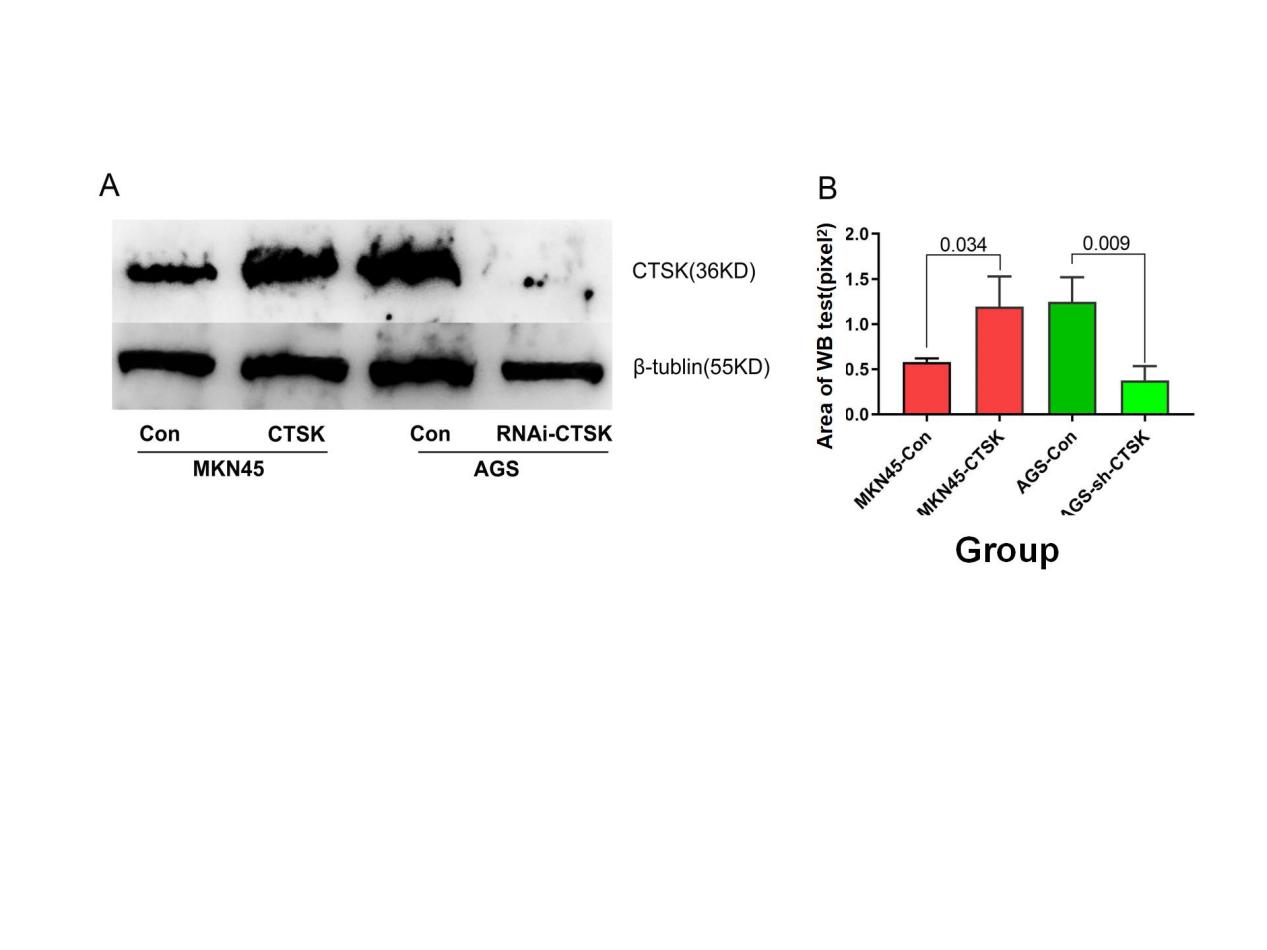


Figure s6 The WB test results of oe- and ko-expressed LV transfected MKN45 and HGC27 cell .
