# Supplementary material for: The potential contribution of aberrant cathepsin K expression to gastric cancer pathogenesis
Source: Discov Oncol. 2024 Jun 10;15:218. doi: 10.1007/s12672-023-00814-z (PMC11164852; doi:10.1007/s12672-023-00814-z)
Supplement: Supplementary file 2 — (DOCX 1570 KB) [file 12672_2023_814_MOESM2_ESM.docx]

Supplementary Material 2

# The overall staining of tissue microarray

## IHC staining


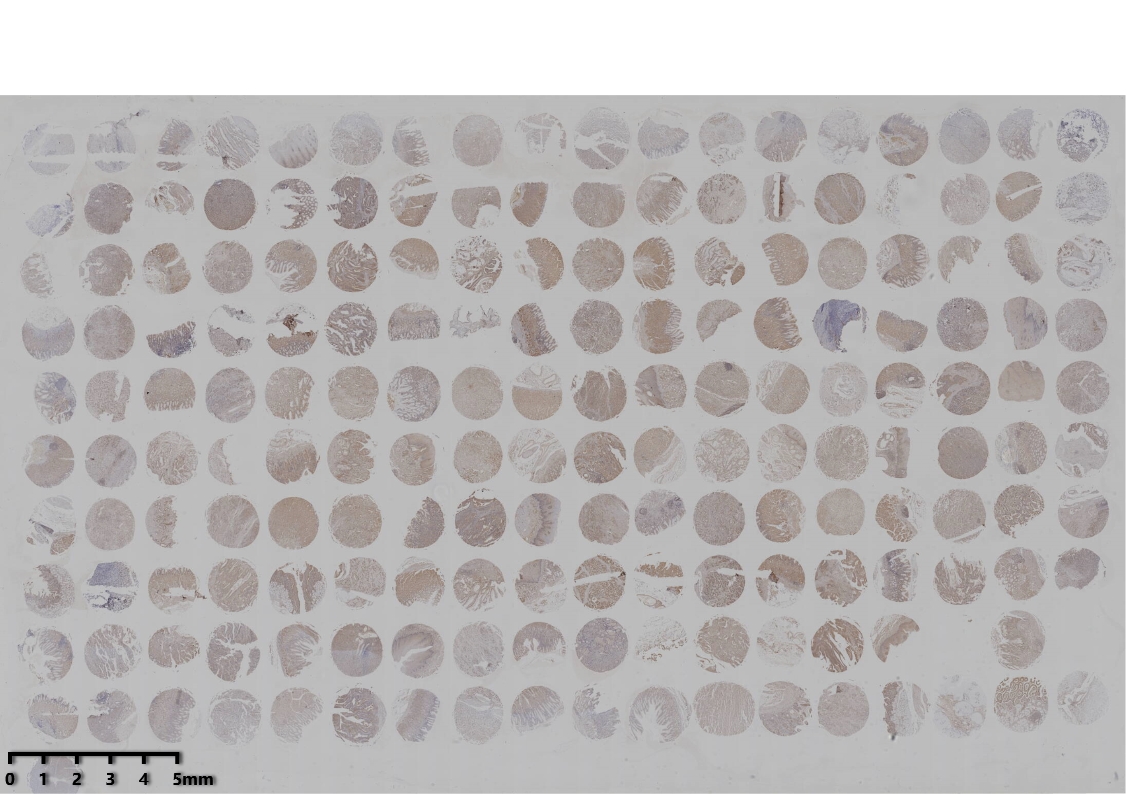
Figure s1 The overall staining of tissue microarray from 90 GC patients


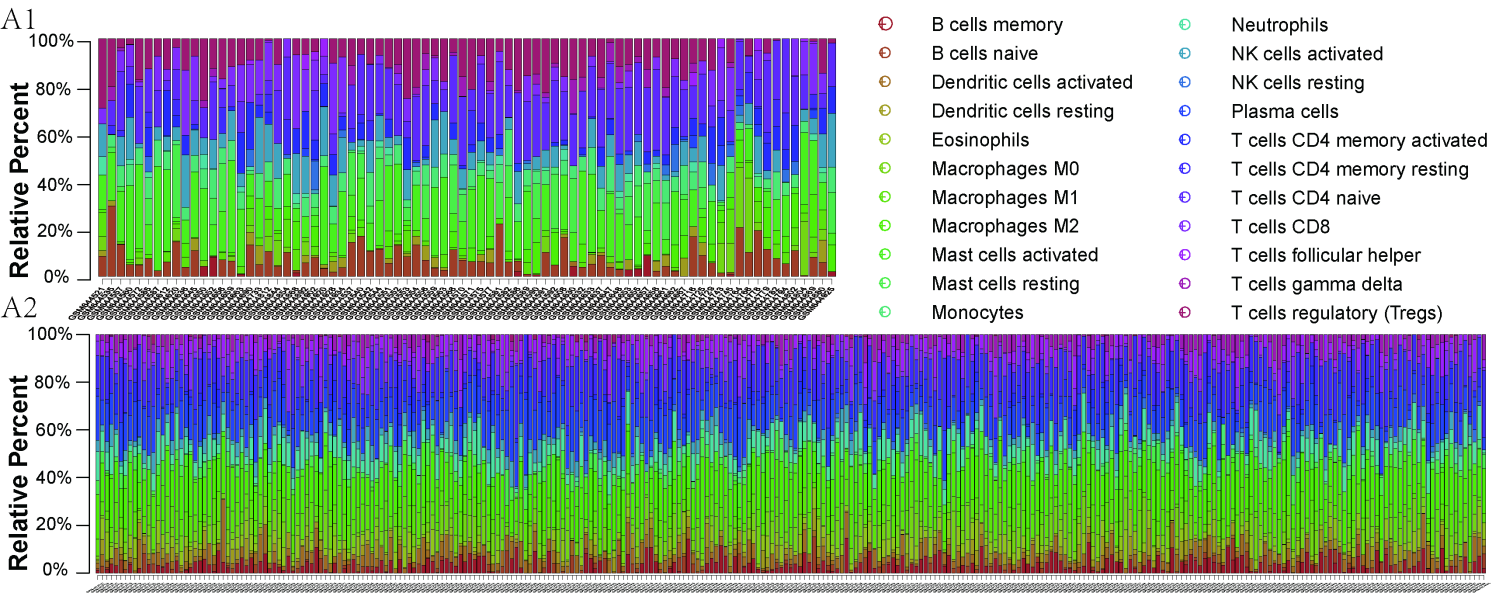
Figure s2 The bar plot illustrating the proportions of the 22 immune cell types from each sample in those two datasets. A1 for GSE26253 and A2 for GSE62254.


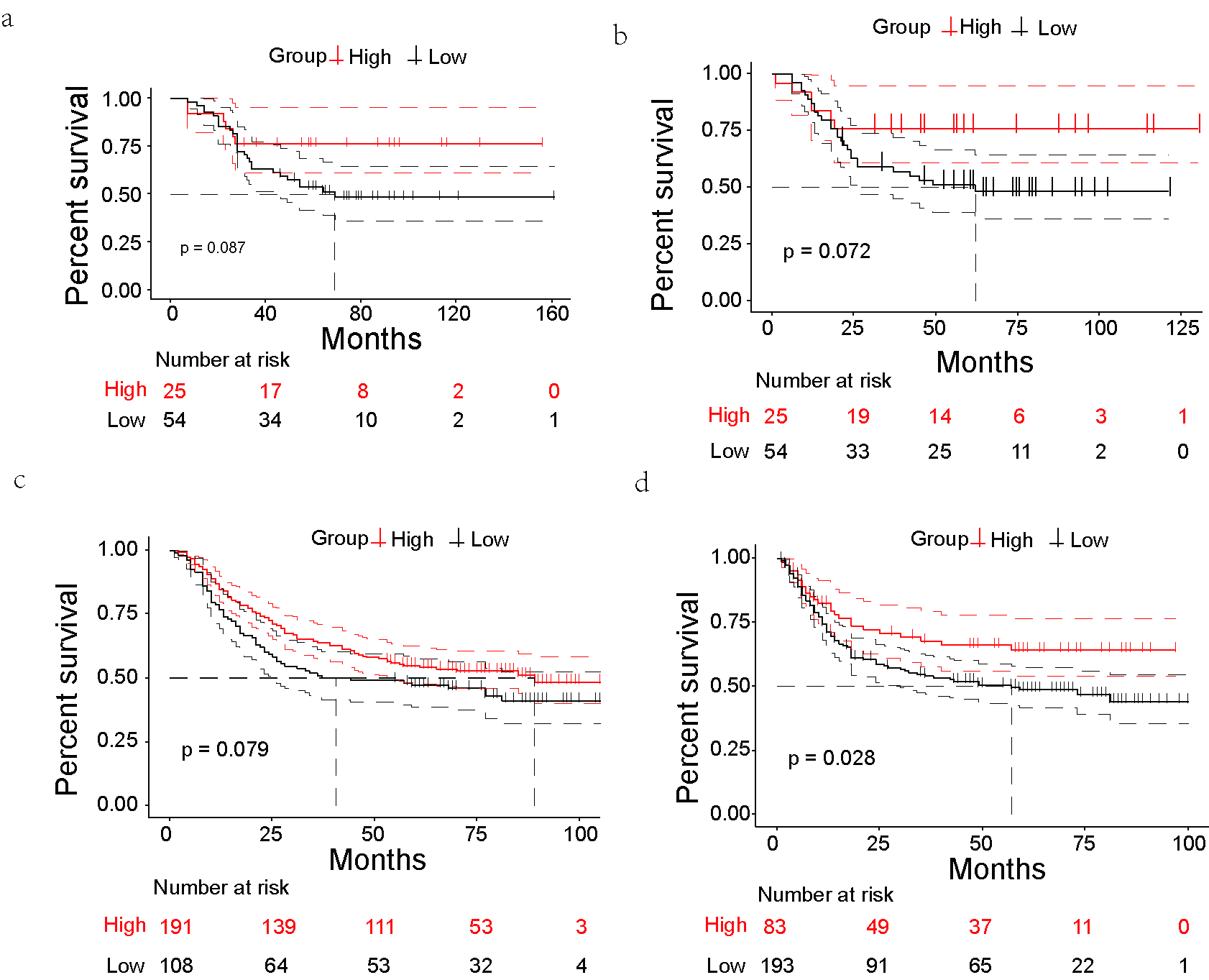


Figure s3 Effect of infiltrating CD8 T cells in the tumor microenvironment on OS and DFS in patients with gastric cancer.a, OS for survival analysis from GSE26253 dataset, b, DFS for survival analysis from GSE26253 dataset, c, OS for survival analysis from GSE62254 dataset, d, DFS for survival analysis from GSE62254 dataset.


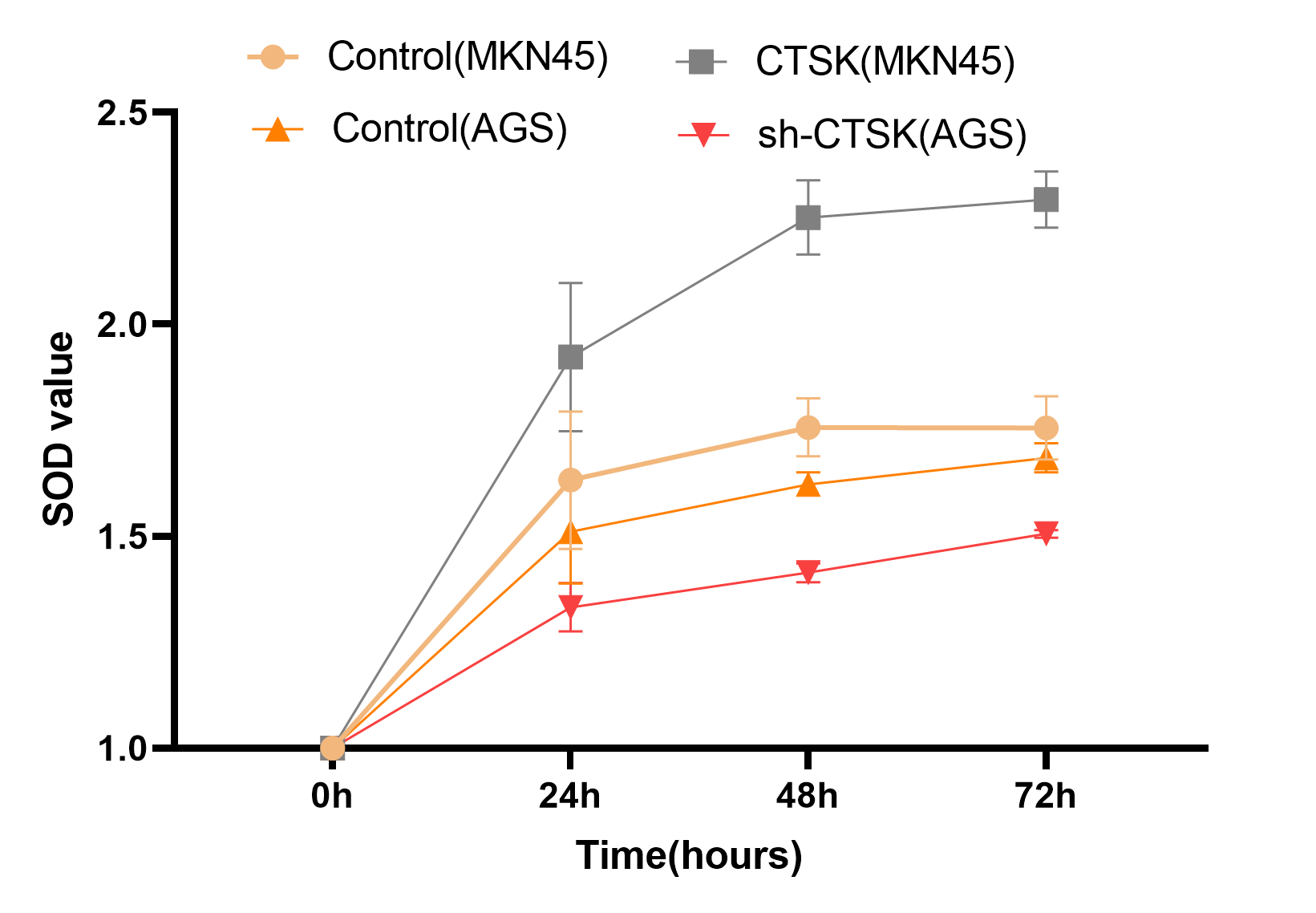
Figure s4 The cell proliferation capacity of the four candidate cell lines was assessed at 24h, 48h, and 72h. MKN45 cell line was over-expressed CTSK, and AGS cell line was knockdown CTSK.
