# Supplementary material for: The potential contribution of aberrant cathepsin K expression to gastric cancer pathogenesis
Source: Discov Oncol. 2024 Jun 10;15:218. doi: 10.1007/s12672-023-00814-z (PMC11164852; doi:10.1007/s12672-023-00814-z)
Supplement: Supplementary file 3 — (DOCX 32 KB) [file 12672_2023_814_MOESM3_ESM.docx]

Supplementary Material

## Original data address

https://www.jianguoyun.com/p/DXbhNZUQ1tfdCxjBu4wFIAA
